# Economic Evaluation of Screening Strategies Combined with HPV Vaccination of Preadolescent Girls for the Prevention of Cervical Cancer in Vientiane, Lao PDR

**DOI:** 10.1371/journal.pone.0162915

**Published:** 2016-09-15

**Authors:** Phetsavanh Chanthavilay, Daniel Reinharz, Mayfong Mayxay, Keokedthong Phongsavan, Donald E. Marsden, Lynne Moore, Lisa J. White

**Affiliations:** 1 Faculty of Postgraduate Studies, University of Health Sciences, Vientiane, Lao PDR; 2 Department of Social and Preventive Medicine, Faculty of Medicine, Laval University, Quebec, Canada; 3 Institut de la Francophonie pour la Médecine tropicale, Vientiane, Lao PDR; 4 Lao-Oxford-Mahosot Hospital-Wellcome Trust Research Unit (LOMWRU), Microbiology Laboratory, Mahosot Hospital, Vientiane, Lao PDR; 5 Centre for Tropical Medicine and Global Health, Churchill Hospital, University of Oxford, Oxford, United Kingdom; 6 Gynecologic Oncology Unit, Setthathirath Hospital, Vientiane, Lao PDR; 7 Mahidol-Oxford Tropical Medicine Research Unit, Faculty of Tropical Medicine, Mahidol University, Bangkok, Thailand; 8 Nuffield Department of Medicine, University of Oxford, Oxford, United Kingdom; Rudjer Boskovic Institute, CROATIA

## Abstract

**Background:**

Several approaches to reduce the incidence of invasive cervical cancers exist. The approach adopted should take into account contextual factors that influence the cost-effectiveness of the available options.

**Objective:**

To determine the cost-effectiveness of screening strategies combined with a vaccination program for 10-year old girls for cervical cancer prevention in Vientiane, Lao PDR.

**Methods:**

A population-based dynamic compartment model was constructed. The interventions consisted of a 10-year old girl vaccination program only, or this program combined with screening strategies, i.e., visual inspection with acetic acid (VIA), cytology-based screening, rapid human papillomavirus (HPV) DNA testing, or combined VIA and cytology testing. Simulations were run over 100 years. In base-case scenario analyses, we assumed a 70% vaccination coverage with lifelong protection and a 50% screening coverage. The outcome of interest was the incremental cost per Disability-Adjusted Life Year (DALY) averted.

**Results:**

In base-case scenarios, compared to the next best strategy, the model predicted that VIA screening of women aged 30–65 years old every three years, combined with vaccination, was the most attractive option, costing 2 544 international dollars (I$) per DALY averted. Meanwhile, rapid HPV DNA testing was predicted to be more attractive than cytology-based screening or its combination with VIA. Among cytology-based screening options, combined VIA with conventional cytology testing was predicted to be the most attractive option. Multi-way sensitivity analyses did not change the results. Compared to rapid HPV DNA testing, VIA had a probability of cost-effectiveness of 73%. Compared to the vaccination only option, the probability that a program consisting of screening women every five years would be cost-effective was around 60% and 80% if the willingness-to-pay threshold is fixed at one and three GDP per capita, respectively.

**Conclusions:**

A VIA screening program in addition to a girl vaccination program was predicted to be the most attractive option in the health care context of Lao PDR. When compared with other screening methods, VIA was the primary recommended method for combination with vaccination in Lao PDR.

## Introduction

While there are few accurate data regarding the incidence or mortality of cervical cancer (or any cancer, in fact) in the Lao PDR, it constitutes a major public health burden with a high rate of morbidity and mortality in both reproductive age and older women [1]. Cervical cancer is the third most common cancer in Lao women and the third leading cause of cancer deaths [1]. The Lao PDR is one of 72 countries eligible for support from the Global Alliance for Vaccines and Immunization (GAVI), for HPV vaccination programs. A pilot project of an HPV vaccination program targeting fifth grade girls was launched in Vientiane capital and Vientiane province in October 2013. Vaccination can be expected to reduce the number of cervical cancers by about 70–75%, by conferring protection against HPV types 16 and 18 related cancers. Moreover, only once high levels of coverage of the female population are achieved, after a few decades of girl vaccination, might unvaccinated women benefit from herd immunity [2]. Therefore large scale community screening programs are needed to significantly affect the incidence and mortality of the disease among the population as a whole [3]. A screening program that targets women who are not covered by the usual vaccination program might be an effective complement to the vaccination of schoolgirls.

Despite the availability of cytology screening facilities in the country, at least in Vientiane Capital, only 5.2% of women aged 18–69 years in urban areas and 1.4% in rural areas have ever had cytological screening, either as part of a community screening program, or opportunistically when visiting a health care facility for some other reason [1]. Opportunistic screening is less effective than organized programs [4], and there are a range of screening strategies which show different levels of efficacy in terms of sensitivity and specificity. Several screening approaches may be implemented. Visual inspection with acetic acid (VIA) and cytology show low reproducibility. Cytology requires expertise, a healthcare infrastructure and resources, and it has a low sensitivity and high cost compared to VIA [5]. Meanwhile, VIA has a low specificity and a low positive predictive value (PPV), leading to unnecessary treatment [6]. In contrast, a rapid HPV DNA testing approach provides simple, accurate and reproducible results [7, 8]. However, its use in developing countries is limited by its high cost and due to the fact that HPV DNA testing only detects HPV infection, but not precursors of cancer, so that there is a need for follow up of positive results [9].

Which screening strategy should be implemented in a developing country with scarce resources devoted to health care like Lao PDR? To answer this crucial question for the country, we need to consider not only the demonstrated effectiveness of a screening program, but also its cost. Health policy decision makers in Lao PDR are lacking key information to decide about the relative value of the diverse screening programs that might be implemented in the country.

The goal of this study was to determine, using mathematical modelling, the cost-effectiveness of various options regarding cervical cancer screening strategies along with an HPV vaccination program for girls in Vientiane, the capital of Lao PDR.

## Materials and Methods

The outcome of interest was incremental cost-effectiveness (C/E) ratios (ICERs). The C/E denominator consisted of 1) the reduction in the incidence of cervical cancers and 2) DALYs averted related to all cervical cancer cases. The numerator consisted of the direct cost of the various options compared, from a public health care system perspective. This economic evaluation study complied with the recommendations of WHO for cost-effectiveness analyses [10].

### Virtual population

The initial virtual population (at year 1) consisted of the entire population of women with characteristics similar to the population of Vientiane capital in terms of age distribution [11] and estimated age-specific incidence rates of cervical cancer in 2014 [1]. The Vientiane capital population was used in the model instead of the whole country due to the fact that the population of the country is predominantly rural [12], and the ethnic mix of the population [13] is likely to be very different in each of the provinces; subsequently the vaccination uptake might be different.

### Model structure and parameters of natural history

The details of the model structure are described in the S1 Appendix. Briefly, a compartmental dynamic population-based model was created to represent the natural history of cervical cancer both in females and males. Susceptible girls and boys were considered to be at risk of being infected based on estimated infection rates between partners. For both males and females, the model considered if the HPV genotype was a 16, 18 or other high-risk types, or if it was of low-risk types.

**Fig 1:**
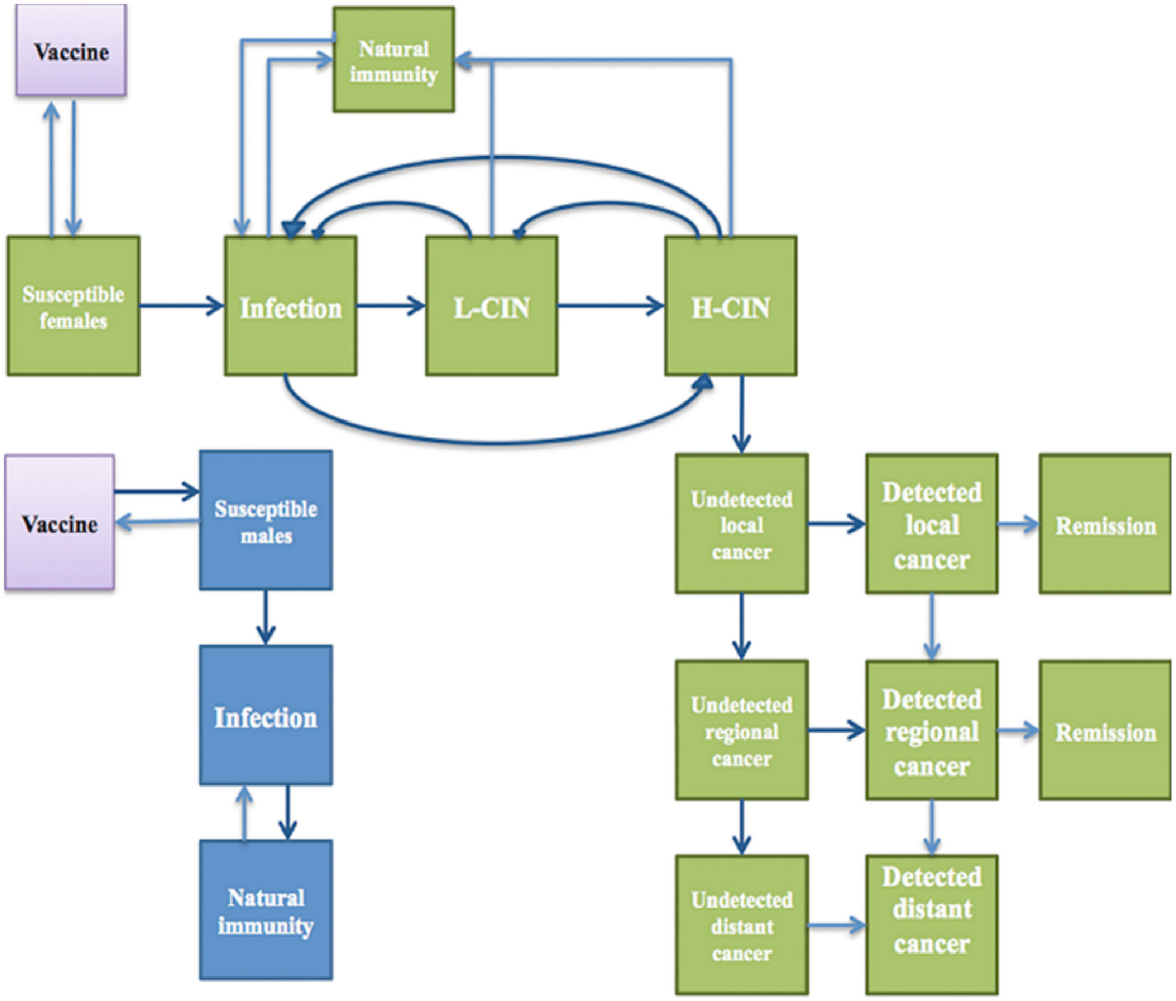
Model structure for the natural history of human papillomavirus infection and cervical cancer. The model structure reflects the natural history of HPV infection towards cervical cancer. Women can be infected by HPV and progress to low-grade CIN or high-grade CIN, or regress with natural immunity. Low-grade CIN progress to high-grade CIN, or regress thanks to the natural immunity. High-grade CIN progress to invasive cervical cancer (local, regional and distant cancer), or regress thanks to the natural immunity. In the male model, there are three compartments considered: susceptibility to infection, infection and recovery with natural immunity. Female can be protected by HPV vaccine.

For females, the model considered that an infection with HPV might recover with natural immunity, while remaining susceptible to infection with other HPV types. HPV infection might persist or progress into a cervical intraepithelial neoplasia (low-grade CIN or high-grade CIN). A low-grade CIN might recover with immunity or regress to an infection state, or progress into a high-grade CIN. A high-grade CIN might recover with immunity or regress to an infection state or low-grade CIN, or might progress into an invasive cervical cancer (local, regional or distant, respectively). Women diagnosed with a high-grade CIN are treated. Women with invasive cervical cancer might be symptomatically detected. Diagnosed cancer cases are treated, with a probability of recovery, treatment failure or death (Fig 1).

For the screening model, true high-grade cervical intraepithelial neoplasia (CIN) cases diagnosed at a cytology examination or rapid HPV DNA testing receive a loop electrosurgical excision procedure (LEEP) or hysterectomy treatment. When VIA is used, a see-and-treat approach is adopted. True positive and false positive high-grade CIN cases receive cryotherapy treatment. The detail of screening procedure is provided in the S1 Appendix. Cured cases regress to a recovery state with type-specific natural immunity. Unscreened or undetected cases, or treatment failure follow the natural history of HPV infection, which may lead to cervical cancer (Fig 1).

For males, only the susceptibility, infection and recovery states were considered. Vaccinated people remained susceptible for non-vaccine HPV types (Fig 1).

The progression and regression rates from one state to another one were based on the literature [14] as described in Table 1. However, we calibrated infection rates and cancer stage-remission rates. The sensitivity and specificity of screening and diagnostic tests and remission rate of precancerous lesions treatment were retrieved from meta-analysis and systematic reviews. Meanwhile, the remission rate of stage-specific invasive cervical cancer was calibrated based on estimates of the mortality related to cervical cancer in Lao PDR (Table 2).

**Table 1:** Summary of input parameters for the natural history of HPV infection and cervical cancer.

Parameters		Baseline values*	Source
**Progression**			
Healthy to infection † (-20 and +40%)	HPV-16	0.000175–0.003148 (0.0001426–0.00761)	Calibrated
	HPV-18	0.0004–0.000789 (0.000102–0.00168)	
	Other HR HPV	0.000206–0.004038 (0.0001703–0.00911)	
	LR HPV	0.000958–0.018412 (0.00069–0.0537)	
HPV DNA to CIN1‡	HR-16 HPV	0.005194–0.00901	[14]
	HR-18 HPV	0.002793–0.004845	
	HR-other HPV	0.007693–0.013345	
	LR-HPV	0.002397–0.001222	
Proportion (%) of women who transition directly from HPV DNA to CIN2,3	HR-16 HPV	0.64	
	HR-18 HPV	0.975	
	HR-other HPV	0.966	
	LR-HPV	0.98	
CIN 1 to CIN 2,3 ‡	HR-16 HPV	0.00951–0.012363	
	HR-18 HPV	0.0051–0.00663	
	HR-other HPV	0.00747–0.009711	
	LR-HPV	0.000149–0.000222	
CIN 2,3 to local cancer	HR-16 HPV	0.000151–0.00906	
	HR-18 HPV	0.000264–0.01584	
	HR-other HPV	0.000199–0.01194	
Local to regional invasive cancer		0.0200	
Regional to distant invasive cancer		0.0250	
**Regression**			
HPV DNA to Normal	HR-16 HPV	0.09089	
	HR-18 HPV	0.09089	
	HR-other HPV	0.09272	
	LR-HPV	0.09699	
CIN 1 to normal ‡‡	HR-16 HPV	0.03782	
	HR-18 HPV	0.03782	
	HR-other HPV	0.04575	
	LR-HPV	0.01708	
CIN 2,3 to Normal §§	HR-16 HPV	0.000798–0.000455	
	HR-18 HPV	0.003556–0.011938	
	HR-other HPV	0.002926–0.009823	
	LR-HPV	0.001904–0.006392	
Other			
Immunity (%) (HR-HPV types only) ¶¶	HR-16 HPV	0.66	
	HR-18 HPV	0.86	
	HR-other HPV	0.59	
Annual probability of symptom detection #	Local invasive cancer	0.33	
	Regional invasive cancer	0.60	
	Distant cancer	0.9	
Proportion of cancer patient receiving the treatment	Local cancer	100%	Assumption
	Regional cancer	87%	
	Distant cancer	78%	
Age-specific 5-year survival proportion after diagnosis and treatment (%) £	Local cancer	0.29–71%	Calibrated
	Regional cancer	0.24–78%	
Age-specific monthly probability of death	Complication of local cancer treatment	0.012–0.037	Calibrated
	Complication of regional cancer treatment	0.0098–0.028	
	Distant cancer (rate)	0.28–0.83	
Age-specific all cause death rates per person per year	Female	0,00106–0,4122	[15]
	Male	0.001–0.47	

* Baseline values are monthly age-specific probabilities, unless otherwise noted

^†^ The transition from healthy state to infection is a force of infection derived from the number of sexual partner change, HPV type-specific transmissibility.

^‡^ HPV, human papillomavirus; CIN, cervical intraepithelial neoplasia; HR, high risk; LR, low risk

^‡‡^ 70% of women with CIN 1 regress to normal, 30% to HPV.

^§§^ 70% of women with CIN2,3 regress to normal, 15% to HPV, 15% to CIN 1.

^¶¶^ Immunity represents the degree to protection each woman faces against future type-specific infection after infection after first infection and clearance. The immunity was assumed to be lifelong.

^#^ The annual probability of symptom detection corresponds to 15% for local cancer and 85% for advanced cancer

^£^ Age-specific survival proportion was calibrate, based on a mortality rate estimated by Globocan [1].

**Table 2:** Summary of input other parameters for the model.

Parameters	Value (range)	Distribution	Source
**VIA**			
Sensitivity (95% Confidence interval)	73.2% (66.5–80.0%)	Beta	[16]
Specificity (95% CI)	86.7% (82.9–90.4%)	Beta	
**Conventional cervical cytology**			
Sensitivity for CIN23	59% (29–82%)	Beta	[17]
Specificity	94% (88–99%)	Beta	
**ThinPrep Cervical cytology**			
Sensitivity for CIN23	88% (70–94%)	Beta	[17, 18]
Specificity	88% (65–97%)	Beta	[19]
**Combined testing VIA and conventional cytology**			
Sensitivity to detect high-grade CIN	87% (83–90%)	Beta	[20]
Specificity	79% (63–89%)	Beta	
**Rapid HPV DNA testing**			
Sensitivity to detect high-grade CIN	81.5% (76.5–85.8%)	Beta	[21]
Specificity	91.6% (81.8%-97.4%)	Beta	
**Colposcopy**			[22]
Sensitivity for high-grade CIN	96% (64–99%)	Beta	
Sensitivity	48% (30–93%)	Beta	
Loss to follow-up per visit	15% (0–50)	Beta	
**Probability of treatment for High grade CIN in Cervical cytology**			
≤ 35 years	LEEP: 80% (50–80%) Hysterectomy: 20% (20–50%)	Beta	Assumption ‡
> 35 years	Hysterectomy: 80% (50–80%) LEEP: 20% (20–50%)	Beta	
**Proportion of recovery**			
Cryotherapy			
Low-grade CIN	94% (85–95)	Beta	[23]
High-grade CIN	86% (83–89)	Beta	
LEEP: High-grade CIN	96.7% (90–98%)	Beta	[24]
Hysterectomy: Any CIN	99% (90–100%)	Beta	[25]
**Access to care**			
Local cervical cancer	80% (50–100)	Beta	Assumption ‡
Regional cervical cancer	80% (0–50)	Beta	Assumption ‡
Distant cervical cancer	80%	Beta	Assumption ‡
Vaccine efficacy against HPV type 16 and 18 infection	100%	Beta	[26]

Note:

^‡^ Assumption was based on experts’ opinion

Women with local cervical cancer are treated by hysterectomy

Women with regional cervical cancer are treated by chemoradiation

Women with distant cancer are given palliative care

### Model calibration

The population was stratified by gender and age. The model is in the form of a realistic age structured (RAS) model. The equations were numerically solved in Berkeley Madonna version 8.3.18 [27]. The model was calibrated using maximum likelihood for the demographic distribution, the age-specific distribution of the 2014-estimated incidence of cervical cancer and mortality related to cervical cancer data in Lao PDR. The demographic distribution followed an exponential distribution using UN data to predict the changing birth and death rates over time for Lao PDR [28]. To calibrate the age-specific incidence of cervical cancer, we assumed that only the infection rate was different from the Kim et al. model [14]. We consequently calculated an infection rate multiplier to calibrate the incidence of cervical cancer according to the Globocan estimates.

The calibration of parameters for the age and stage-specific mortality rates of cervical cancer was conducted by varying the proportion of women receiving treatment for local, regional and distant cancer, the monthly death rates due to treatment complications and the age and stage-specific remission rates. The true proportion of women receiving a treatment in Lao PDR is unknown; we therefore estimated its value according to the experts’ opinion. The best guess of the proportion of women receiving a treatment for a local, regional or distant cancer was 100%, 80% and 70%, respectively.

### Scenarios

Scenarios included the baseline option and the various prevention program options. The baseline option considered that no vaccination program existed and that, based on WHO estimates, the coverage of a cytology screening program where screening was repeated every three years was 5.2% [1].

The scenarios were built on the following options: 1) girls vaccination alone, 2) girls vaccination combined with screening: either with VIA, rapid HPV DNA testing, combined VIA and conventional cytology testing, liquid-based cytology (LBC) or conventional cytology, and 3) screening alone. The combined VIA and conventional cytology is giving VIA and cytology testing at the same time. The positivity is defined as either one positive. Screening strategies were selected based on feasibility and accessibility considerations relevant to the Lao context. Screening, VIA, cytology and HPV DNA testing, are done by a ynaecologica during ynaecological outpatient visits.

In each screening scenario, different initial ages for screening were considered: 20, 25 and 30 years old. The maximum age was fixed at 65 years. The initial age of screening was considered in three different group in comparison. Starting screening at 20 years of age is the recommendation for HPV DNA testing in USA [29], 30 years of age as a recommendation of WHO [30]. Moreover, the frequency of screening was fixed at either yearly, three-yearly or five-yearly intervals. Yearly interval screening reflects the current practice in Vientiane Capital. Three-yearly intervals follow WHO recommendations [30] and five-yearly intervals are current practice in the USA [29].

In all options, base-case analyses were performed with a screening coverage assumed to be 50% (range: 10%-80%). Lost to follow-up was assumed to be 15% per visit (range: 0%-50%). The proportion of women receiving cancer treatment among diagnosed patients was calibrated to the mortality rate of cervical cancer. The age- and stage-specific monthly remission rates for cancer treatment were calibrated based on the estimated mortality rates of cervical cancer (summary table in S1 Appendix) [1]. We assumed that the cytology alone option or combined with VIA is a three-visit approach. The first visit refers to primary screening; the second refers to receiving the results and making an appointment in case of a positive result. The third refers to colposcopy with direct biopsy. Meanwhile, rapid HPV DNA testing is a two-visit approach. The first visit refers to primary screening. The second refers to colposcopy with direct biopsy in case of a positive result. VIA is considered as a single-visit “see-and-treat approach”.

The coverage of HPV vaccination in girls was assumed to be about 70% (30–80%), with 100% (30–100%) effectiveness against HPV types 16 and 18 and a lifelong protection (10 years to lifelong).

### Costing

We considered a public health care system perspective for cost estimations. Only direct medical and programmatic costs were considered. Details on the approach used to calculate the consumption of items are described in the S1 Appendix. Briefly, we used data from the Ministry of Health (personal communication with Maytry Senchanthixay, 2014) collected in central hospitals for a study aimed at determining costs per patient for each hospital. These data were used to estimate the screening visit and treatment cost. The cytology alone or combined with VIA options requires three visits. The first visit is for screening, the second for receiving the result and making an appointment for positive cases. The third is for a colposcopy with a direct biopsy. Meanwhile, rapid HPV DNA testing requires two visits. The first is for primary screening, the second for a colposcopy with direct biopsy in the case of a positive result. VIA requires only one “see-and-treat approach” visit.

The cost of invasive cervical cancer treatment was retrieved from a study done in 72 GAVI-eligible countries [31]. The items of the screening programmatic cost included quality control, training, administration and recruitment costs.

The base case per dose cost of the vaccine was based on the purchasing cost from GAVI (4.5 I$ per dose) [32]. The full programmatic cost of the three-dose HPV vaccine per girl is 29.1 international dollars (I$) according to a previous survey on the pilot project of girl vaccination in Vientiane capital (Personal communication with Phanmanysone Philakong, 2015). Unit prices are reported as 2013 international dollars, using the purchasing power parity (PPP) exchange rate (1 international dollar I$ = 2,694.27 Lao kip) [33]. To converse the local currency to international dollars, tradable and non-tradable goods were separately conversed (Table 3).

**Table 3:** Costing parameters.

Item	Unit price (International dollar)	Distribution	Source
VIA ‡	26.45	Gamma	Personal communication with a head of department of health insurance. Ministry of health, Lao PDR
Conventional cervical cytology ‡	48.27	Gamma	
Liquid-based (Thin-Prep) cervical cytology ‡	64.21	Gamma	
VIA+ Conventional cervical cytology	50.91	Gamma	
Rapid test of HPV DNA testing ‡	47.18	Gamma	
Colposcopy	17.87	Gamma	
Cryotherapy	23.59	Gamma	
LEEP	120.40	Gamma	
Hysterectomy	1188.59	Gamma	
Treatment cost of Local cancer §§	745.57 (372.79–1491.15)	Gamma	[31]
Treatment cost of regional cancer §§	845.68 (422.85–1691.36)	Gamma	
Treatment cost of distant cancer §§	845.68 (422.85–1691.36)	Gamma	
Vaccine cost per doses	4.5	Gamma	[32]
Programmatic cost of vaccination for three doses	29.1	Gamma	Personal communication with Phanmanysone Philakong, WHO.

Note:

^‡^ Screening cost includes both direct medical cost and programmatic cost.

^§§^ Cost is unit price per person, 2013 International dollars exchange using purchasing power parity (PPP) exchange rate (1 I$ = 2,694.27 kips) [33] and the price of cancer treatment was adjusted from 2005 to 2014 using consumer price index (77.33 in 2005 and 122.52 in 2014) [11]

### Simulation analyses

The simulation process was run deterministically over a 100 year-span to capture the short and long term benefits of vaccination. For each option, the output consisted of the cumulative number of cervical cancers per 1 000 women, the DALYs per 1 000 women, and the cost of screening and treatment per 1 000 women. The strategies were ranked based on their cost, from the lowest to the highest. In the case of a non-dominant situation, strong or extended dominance, the incremental cost-effectiveness ratio was calculated using the reduction of cervical cancer cases and DALYs averted as denominators. DALYs were calculated based on the Global Burden of Disease without age weighting [34]. The disability weight for cancer treatment was retrieved from the current literature [35]. For each strategy, C/E was calculated using the reduction in the number of cervical cancer cases and DALYs averted as denominators.

All costs and DALYs were discounted at a rate of 3% in base case simulations to convert future costs and life expectancies and durations of disability to their present value [10]. However, other discount rates of 0% to 5% for DALYs and 6% for costs were also explored. The results were interpreted taking into account the recommendations of the UN Commission on Macroeconomics and Health which proposed classifying cost-effectiveness studies into three categories: 1) highly cost-effective (ICER<GDP per capita); 2) cost-effective (ICER between 1–3 times GDP per capita); and 3) not cost-effective (ICER>3 times GDP per capita), based on the willingness-to-pay threshold recommended by the UN Commission on Macroeconomics and Health [36]. The GDP per capita in 2013 was about 4,822 international dollars using the Purchasing Power Parity (PPP) exchange rate [37].

### Sensitivity analyses

One-way sensitivity analyses were conducted on the cost of the vaccine, screening and vaccination coverage, lost to follow-up, and sensitivity of VIA and conventional cytology, which were expected to significantly influence the incremental cost-effectiveness ratios.

In order to take into account uncertainties and joint effects, multi-way sensitivity analyses on parameters were conducted using a probabilistic sensitivity analysis. Each parameter was randomly drawn from its distribution (summary table in the S1 Appendix). As stated above, the parameters that were varied included: natural history progression of HPV infection, the proportion of people receiving treatment, monthly remission rates of precancerous lesions and cancer treatment, screening sensitivity and specificity, screening coverage, vaccination coverage, waning of natural and vaccine-induced immunity, effectiveness of the vaccine, disability weight, and discount rate. The costing parameters, with the exception of cancer treatment, were varied by 75% in the sensitivity analyses (summary table in the S1 Appendix) by using gamma distributions. A lognormal distribution was used for the multipliers of the natural history of cervical cancer and a beta distribution for other parameters.

The Monte Carlo simulation was run for 1 000 iterations with Berkeley Madonna [27]. The program computes mean and standard deviations for each option. Acceptability curves were produced according to the probability of the ICERs to be cost-effective, taking into account the recommendation of the UN Commission on Macroeconomics and Health on various ceiling ratios. Acceptability curves were produced to take into account various willingness-to-pay thresholds as recommended by the UN Commission on Macroeconomics and Health [36]. The acceptability curve was based on the results of probabilistic sensitivity analyses [38].

## Results

### Impact of prevention strategies

The model output of demographic distribution showed that the virtual population was similar to the general population in terms of age distribution and trends over time. After the equilibrium state was reached, the age-specific incidence of cervical cancer and the mortality rate predicted were similar to expected values, and were consistent over the time span of the simulation (S1 Appendix).

In base-case analyses, the most effective strategy was a program consisting of annual VIA screening for 20–65 year old women in addition to a vaccination program for 10-year-old girls. This strategy was predicted to prevent 87% of cervical cancers and produce a gain of 50 DALYs per 1 000 women, about 32% less cancer and 11 additional averted DALYs per 1 000 women compared to a program consisting of only vaccination in girls. In the case that implementing a VIA program is not realistic, rapid HPV DNA testing in addition to a vaccination program was predicted to be the most effective option, with an 85.7% cancer reduction and 49.3 DALYs averted per 1 000 women. Among cytology-based screening strategies, LBC and combined VIA and cytology testing in addition to a vaccination program were predicted to be equally the most effective options, with an 84% cancer reduction and 49 DALYs averted per 1 000 women.

When we compared different initial ages for a screening program and frequencies of screening within the same screening strategy, we found that screening at an early age of 20 or 25 years old, added a slight reduction in the number of cancers compared to a program starting at the age of 30. The number of cancers was predicted to be further reduced when the frequency of the screening intervals was increased, i.e. screening with VIA for 30–65 year old women alone was shown to reduce the number of cancers by 44%, 58% and 80%, if screening was performed every five years, every three years or every year, respectively.

In terms of costs, LBC was predicted to be the most expensive option, followed by the combined VIA and cytology testing, rapid HPV DNA testing and VIA options, respectively (S1 Appendix).

### Cost-effectiveness

Table 4 shows the comparison of all available screening strategies. In base-case scenarios, the girl vaccination only program was dominated by a program consisting of three-yearly VIA screening for 30–65 year old women. VIA screening also dominated other screening strategies. Therefore, comparisons were conducted only among VIA screening options. Two strategies, based on the GDP per capita threshold ratios, were predicted to be very cost-effective in terms of cancer reduction compared to the next best strategy: a program consisting of VIA screening for 30–65 year old women every five years alone, or an every-three-year program combined with vaccination. These cost 4 468 I$ and 4 166 per case cancer reduction and 2 544 I$ and 351 I$ per DALY averted, respectively. In addition to these strategies, others were also predicted to be very cost-effective in terms of DALYs averted. These included a VIA screening program targeting 25–65 year old women every five years, a VIA screening program targeting 30–65 year old women every three years alone and a VIA screening program targeting 30–65 year old women every five years to which is added a girl vaccination component. These cost 856 I$; 1 064 I$; and 1 362 I$ per DALYs averted, respectively.

**Table 4:** Cost-effectiveness of screening strategy alone combined with 10-years-old girl vaccination.

Option	Total cost per 1000 women	Cancers per 1000 women	Cancer reduction (%)	DALYs averted per 1000 women	ICER (cancer reduction)	ICER (DALYs averted)
**All screenings are realistic**						
Baseline	4716	4.8	Ref	Ref	-	-
Five-yearly VIA_30–65	13325	2.7	43.5	24.5	4166	351
Five-yearly VIA_25–65	15598	2.5	47.7	27	11302	895
Three-yearly VIA_30–65	21766	2	57.9	32.8	12771	1064
Vaccination	21824	2.1	54.9	30.7	D	D
Five-yearly VIA_30–65 + vaccination	30577	1.4	69.7	39.3	15718	1362
Five-yearly VIA_25–65 + vaccination	32862	1.4	70.5	39.8	D	ED
Five-yearly VIA_20–65 + vaccination	35202	1.4	71	40.2	ED	ED
Three-yearly cytology_30–65	37199	3	36.6	21.8	D	D
Three-yearly HPV testing_30–65	37242	2.2	53.5	31.8	D	D
Three-yearly VIA_30–65 + vaccination	39051	1.2	75.2	42.6	4468	2544
Three-yearly VIA+cytology_30–65	41858	2.5	48.3	28.7	D	D
Three-yearly VIA_25–65 + vaccination	42862	1.1	76.1	43.2	D	ED
Three-yearly VIA_20–65 + vaccination	46763	1.1	76.7	43.6	D	ED
Five-yearly HPV testing_20–65 + vaccination	47694	1.4	69.6	40.2	ED	ED
Three-yearly LBC_30–65	49868	2.4	48.6	28.9	D	D
Three-yearly cytology_30–65 + vaccination	54264	1.6	67.1	38.5	D	D
Three-yearly HPV testing_30–65 + vaccination	54327	1.3	73.5	42.6	D	D
Three-yearly VIA+cytology_30–65 + vaccination	58935	1.4	71.5	41.3	D	D
Three-yearly HPV testing_25–65 + vaccination	60775	1.2	74.4	43.1	ED	ED
Yearly VIA_30–65	64261	1	79.9	45.7	ED	ED
Three-yearly LBC_30–65 + vaccination	66944	1.3	71.6	41.4	D	D
Three-yearly HPV testing_20–65 + vaccination	67411	1.2	75	43.5	D	D
Yearly VIA_30–65 + vaccination	81575	0.7	85.7	49	85116	6733
Yearly VIA_25–65 + vaccination	93002	0.6	86.5	49.4	D	24136
Yearly VIA_20–65 + vaccination	104683	0.6	87	49.8	422480	30462
Yearly HPV testing_30–65	109208	1.1	77.5	45.7	D	D
Yearly cytology_30–65	109312	1.6	66.5	39.3	D	D
Yearly VIA+cytology_30–65	123124	1.2	74.5	44	D	D
Yearly HPV testing_30–65 + vaccination	126370	0.7	84.4	49.2	D	D
Yearly cytology_30–65 + vaccination	126424	1	78.8	45.8	D	D
Yearly VIA+cytology_30–65 + vaccination	140273	0.8	82.8	48.2	D	D
Yearly HPV testing_25–65 + vaccination	145701	0.7	85.1	49.6	567338	42121
Yearly LBC_30–65	147137	1.2	74.7	44.1	D	D
Yearly LBC_30–65 + vaccination	164287	0.8	82.9	48.2	D	D
Yearly HPV testing_20–65 + vaccination	165588	0.7	85.7	49.6	D	D

Note:

All screening strategies with different initial age “20, 25, and 30 years old” and screening interval “every year, and” were analyzed, but only some are presented here in this table. The detail is described in Appendix.

Baseline refers to no vaccination with 5.2% cytology screening for women aged 18–68 years old.

Vaccination is for 10-years-old girls. Cytology refers to conventional cervical cytology; LBC refers to liquid-based cervical cytology; HPV testing refers to rapid HPV DNA testing; VIA+cytology refers to the combined testing VIA and cytology.

The incremental cost of effectiveness ratio expressed as cancer prevented or DALY averted is listed in order of increasing cost. In non-dominant strategy, the ICER was calculated by devising different cost to different effectiveness.

**D** refers to strong dominance, which is expressed as higher cost, but lower effectiveness than alternative options.

**ED** refers to extendedly dominance, which has higher ICER than the next ICER.

If VIA alone is not realistic, rapid HPV DNA testing was predicted to be more cost-effective than cytology-based screening. Among these options, compared to the next best strategy, a screening program for 30–65 year old women every 3 and 5 years in addition to a girl vaccination component were predicted to be very cost-effective. They cost 4 391 and 2 102 I$ per DALYs averted. An annual screening strategy was predicted to be cost-effective, costing 10 983 I$ per DALYs averted.

If cytology and the combined VIA and conventional cytology testing strategies are realistic, the combined VIA with conventional cytology testing option was predicted to be more cost-effective than the cytology-based screening alone option. Among these, a program consisting of screening 30–65 year old women every five years was the most attractive option, costing 2 836 I$ per DALY averted compared to a program consisting of only girl vaccination. Regarding the cytology-based screening options, we found that a girl vaccination program dominated screening alone options. LBC was predicted to be more cost-effective than conventional cytology. A program consisting of screening 30–65 year old women every five years in addition to a girl vaccination program was the most attractive option, costing 3 455 I$ per DALY averted compared to the vaccination alone option. Also, the conventional cytology-based screening option using a five-year interval was predicted to be more cost-effective than using other frequency intervals (Table 5).

**Table 5:** The incremental cost effectiveness ratio (ICER) of screening strategies and 10-year-old girl vaccination by realistic assumption.

VIA alone is not realistic	ICER (cancer reduction)	ICER (DALY averted)	When cytology or combined with VIA is realistic	ICER (cancer reduction)	ICER (DALY averted)
Baseline	-	-	Baseline	-	-
Vaccination	6555	557	Vaccination	6555	557
Five-yearly cytology_30–65	D	D	Five-yearly cytology_30–65	D	D
Five-yearly HPV testing_30–65	D	D	Five-yearly VIA+cytology_30–65	D	D
Five-yearly VIA+cytology_30–65	D	D	Five-yearly LBC_30–65	D	D
Five-yearly LBC_30–65	D	D	Three-yearly cytology_30–65	D	D
Three-yearly cytology_30–65	D	D	Five-yearly cytology_30–65 + vaccination	ED	ED
Three-yearly HPV testing_30–65	ED	ED	Three-yearly VIA+cytology_30–65	D	D
Five-yearly cytology_30–65 + vaccination	ED	ED	Five-yearly VIA+cytology_30–65 + vaccination	38253	2836
Five-yearly HPV testing_30–65 + vaccination	28397	2102	Five-yearly VIA+cytology_25–65 + vaccination	ED	ED
Three-yearly VIA+cytology_30–65	D	D	Five-yearly LBC_30–65 + vaccination	D	D
Five-yearly VIA+cytology_30–65 + vaccination	D	D	Three-yearly LBC_30–65	D	D
Five-yearly LBC_30–65 + vaccination	D	D	Five-yearly VIA+cytology_20–65 + vaccination	ED	ED
Three-yearly LBC_30–65	D	D	Three-yearly cytology_30–65 + vaccination	D	D
Three-yearly cytology_30–65 + vaccination	D	D	Three-yearly VIA+cytology_30–65 + vaccination	66830	5068
Three-yearly HPV testing_30–65 + vaccination	57639	4391	Three-yearly VIA+cytology_25–65 + vaccination	ED	ED
Three-yearly VIA+cytology_30–65 + vaccination	D	D	Three-yearly LBC_30–65 + vaccination	D	D
Three-yearly LBC_30–65 + vaccination	D	D	Three-yearly VIA+cytology_20–65 + vaccination	ED	ED
Yearly HPV testing_30–65	ED	ED	Yearly cytology_30–65	D	D
Yearly cytology_30–65	D	D	Yearly VIA+cytology_30–65	ED	ED
Yearly VIA+cytology_30–65	D	D	Yearly cytology_30–65 + vaccination	ED	ED
Yearly HPV testing_30–65 + vaccination	139597	10983	Yearly VIA+cytology_30–65 + vaccination	151018	11771
Yearly cytology_30–65 + vaccination	D	D	Yearly LBC_30–65	D	D
Yearly VIA+cytology_30–65 + vaccination	D	D	Yearly VIA+cytology_25–65 + vaccination	608081	44987
Yearly LBC_30–65	D	D	Yearly LBC_30–65 + vaccination	D	D
Yearly LBC_30–65 + vaccination	D	D	Yearly VIA+cytology_20–65 + vaccination	786975	61537
**When only cytology is realistic**			**When only conventional cytology is realistic**		
Baseline	-	-	Baseline	-	-
Vaccination	6555	557	Vaccination	6555	557
Five-yearly cytology_30–65	D	D	Five-yearly cytology_30–65	D	D
Five-yearly LBC_30–65	D	D	Five-yearly cytology_25–65	D	D
Three-yearly cytology_30–65	D	D	Five-yearly cytology_20–65	D	D
Five-yearly cytology_30–65 + vaccination	ED	ED	Three-yearly cytology_30–65	D	D
Five-yearly LBC_30–65 + vaccination	46610	3455	Five-yearly cytology_30–65 + vaccination	49716	3709
Three-yearly LBC_30–65	D	D	Three-yearly cytology_25–65	D	D
Five-yearly LBC_25–65 + vaccination	D	ED	Five-yearly cytology_25–65 + vaccination	D	ED
Three-yearly cytology_30–65 + vaccination	D	D	Five-yearly cytology_20–65 + vaccination	D	ED
Five-yearly LBC_20–65 + vaccination	D	ED	Three-yearly cytology_20–65	D	D
Three-yearly LBC_30–65 + vaccination	79743	6048	Three-yearly cytology_30–65 + vaccination	66648	5017
Three-yearly LBC_25–65 + vaccination	D	D	Three-yearly cytology_25–65 + vaccination	D	ED
Three-yearly LBC_20–65 + vaccination	ED	ED	Three-yearly cytology_20–65 + vaccination	ED	ED
Yearly cytology_30–65	D	D	Yearly cytology_30–65	D	D
Yearly cytology_30–65 + vaccination	172755	13544	Yearly cytology_30–65 + vaccination	128937	9888
Yearly LBC_30–65	D	D	Yearly cytology_25–65	D	D
Yearly LBC_30–65 + vaccination	196119	15155	Yearly cytology_25–65 + vaccination	490402	35960
Yearly LBC_25–65 + vaccination	730436	54053	Yearly cytology_20–65	D	D
Yearly LBC_20–65 + vaccination	6611733	73818	Yearly cytology_20–65 + vaccination	D	51006

Note:

All screening strategies with different initial age “20, 25, and 30 years old” and screening interval “every year, and” were analysed, but only some are presented here in this table.

Baseline refers to no vaccination with 5.2% cytology screening for women aged 18–68 years old.

Vaccination is for 10-years-old girls. Cytology refers to conventional cervical cytology; LBC refers to liquid-based cervical cytology; HPV testing refers to rapid HPV DNA testing; VIA+cytology refers to the combined testing VIA and cytology.

The incremental cost of effectiveness ratio expressed as cancer prevented or DALY averted is listed in order of increasing cost. In non-dominant strategy, the ICER was calculated by devising different cost to different effectiveness.

**D** refers to strong dominance, which is expressed as higher cost, but lower effectiveness than alternative options.

**ED** refers to extendedly dominance, which has higher ICER than the next ICER.

### Sensitivity analyses

One-way sensitivity analyses showed that three-yearly VIA alone for women aged 30–65 years old is less effective than the HPV vaccination alone when its coverage is lower than 50% or when its sensitivity is 58.5% and lower. As a result, HPV vaccination alone becomes more cost-effective than the three-yearly VIA alone in this case. The on-way sensitivity analyses also showed that it was still more cost-effective to combine the screening strategy with vaccination than either component alone when the vaccination coverage was suboptimal. The same result was found when screening coverage was suboptimal, compared to vaccination alone. However, the combination of screening and vaccination was predicted to provide ICER higher than one GDP per capita when the cost of vaccine is higher than 50 I$ per dose. Meanwhile, rapid HPV DNA testing became more attractive when the sensitivity of VIA was 30% less than the one in the base-case or when there was no loss to follow-up for rapid HPV DNA testing screening. Also, LBC was not more cost-effective than conventional cytology if the sensitivity of conventional cytology was 70% or higher (S1 Appendix).

Multi-way sensitivity analyses did not change the rank of cost-effectiveness in base-case analyses. VIA screening remained dominant over other screening methods, but screening 30–65 year old women with VIA every year becomes more attractive than every three years, with an average cost of 4 202 I$ per DALY averted. Meanwhile, the ICER for a combined vaccination and VIA screening every three years was 1 567 I$ per DALY averted compared to screening every five years.

**Fig 2:**
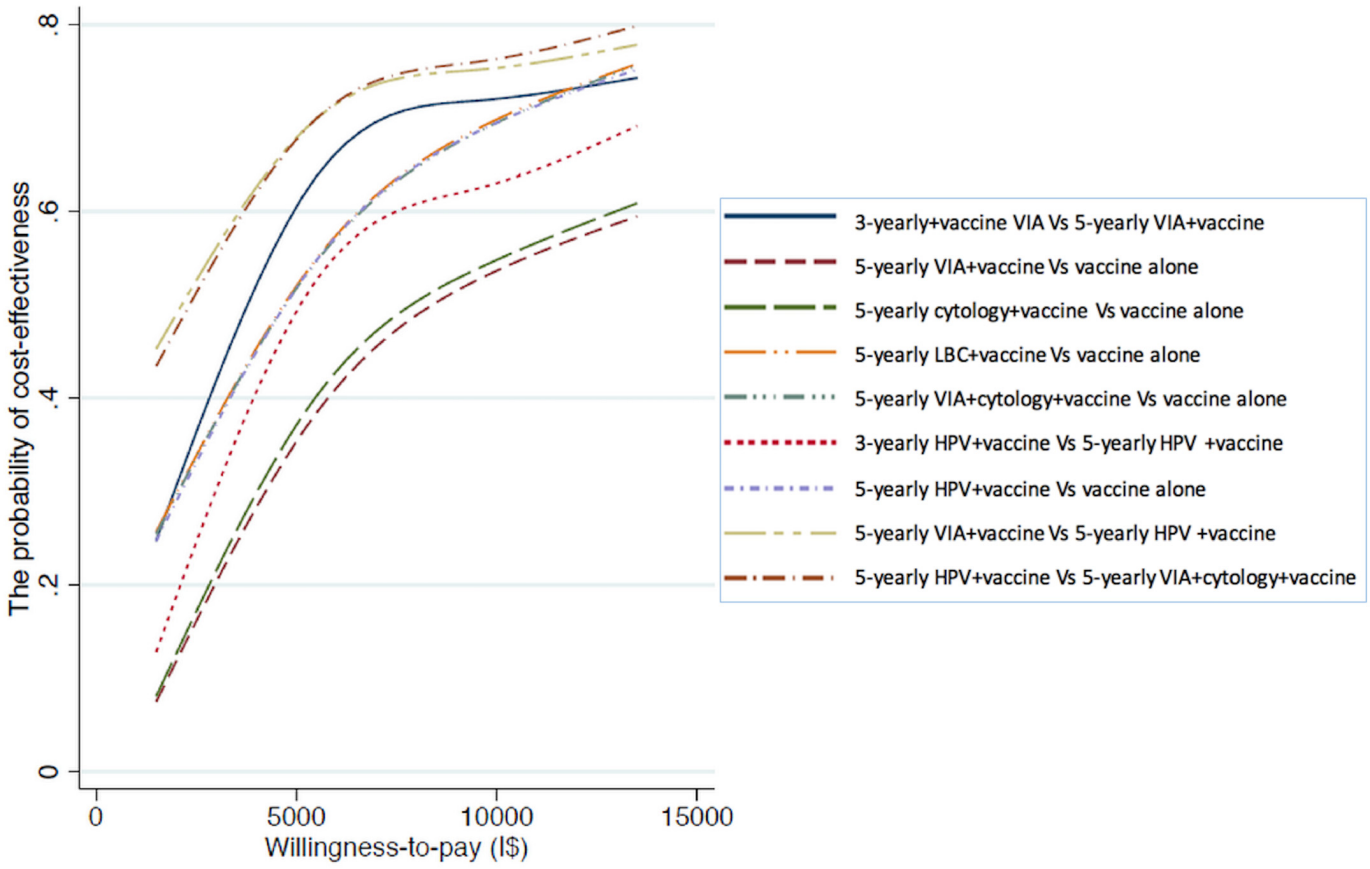
The probability of cost-effectiveness of combined vaccination and screening by willingness-to pay. Note: All screenings stated above are combined with girl vaccination. Excepted where is noted, the five-yearly screenings are compared to vaccination alone. For three-yearly screening is compared to five-yearly one in the same screening technique. LBC refers to liquid-based cytology. VIA+cytology is a combined testing VIA and cytology at the same time. HPV refers to rapid HPV DNA testing.

Fig 2 shows the probability of cost-effectiveness for combined vaccination and screening strategies compared to vaccination alone or different screening intervals. Among these, the probability of cost-effectiveness for the three-yearly VIA screening program for 30–65 years old women was about 67% compared to a five-yearly VIA screening. When this strategy was compared to rapid HPV DNA testing, the probability of cost-effectiveness became 73%. Meanwhile, compared to vaccination alone, the probability of cost-effectiveness for a five-yearly rapid HPV DNA testing was similar to a five-yearly combined VIA and cytology testing and LBC, about 60%. When willingness-to-pay increased to 3 times GDP per capita, probability increased to around 80%, with the exception of the conventional cytology-based screening option, which was about 60%.

## Discussion

Four main findings can be deduced from our results: 1) the combined vaccination and screening option was predicted to be more cost-effective than either strategy alone; 2) screening 30–65 year old women with VIA every three years in addition to girl vaccination was the most attractive strategy; 3) excluding the VIA screening alone option, rapid HPV DNA testing in addition to a girl vaccination program was predicted to be the most attractive option, followed by combined VIA and conventional cytology testing, the liquid-based cytology and conventional cytology option in addition to a girl vaccination program option, respectively; 4) the probability of cost-effectiveness was around 60–67% for screening strategies.

In base-case analyses, the option three-yearly VIA screening for women aged 30–65 years old dominated the girl HPV vaccination alone option. Our findings are different from what has been shown by others. This might be explained by the fact that most studies have been conducted in developed countries where VIA screening was not considered. As found in our study, rapid HPV DNA testing and cytology alone are less effective than HPV vaccination. Furthermore, among studies that included the VIA screening option, different strategies such as VIA screening once or five times in a lifetime and a lower screening coverage were considered [39]. Moreover, Praditsitthikorn and colleagues [40] assumed 20% screening coverage. In our study, once, the screening coverage is less than 50% or the sensitivity of VIA is equal or less than 58.5%, the HPV vaccination option becomes more cost-effective.

Among screening strategies, VIA screening was predicted to be the most attractive option. This result is similar to that of other studies conducted in developing countries [40–42]. This might be explained by the combination of its advantages. First, VIA screening has higher sensitivity, in base case scenarios, than a conventional cervical cytology option and only slightly lower than other screening options considered in this study. Second, VIA can be used as a single-visit approach; subsequently there is no loss to follow-up among positive cases that require treatment. Third, although cryotherapy, the treatment for VIA positive patients, who may have either low-grade or high-grade precancerous lesions, has a slightly higher recurrence rate [23] compared to the loop electrosurgical excision procedure (LEEP), the difference is so small that it would not be expected to impact the overall effectiveness of treatment for precancerous lesions. Nevertheless, VIA is controversial due to its limitations; a positive VIA does not always reflect precancerous or cancerous lesions. Furthermore, invasive cervical cancer cases might not be adequately treated on the basis of a VIA result alone [43]. VIA has a low positive predictive value, which could lead to unnecessary treatment and psychological repercussions [6, 44]. A positive VIA result can be due to polyps, inflammatory conditions, or squamous metaplasia [45]. Also, VIA is subjective. Its interpretation requires careful training and supervision. It is also not appropriate for postmenopausal women because lesions may occur within the endocervical canal, which cannot be visualized. That is why WHO recommends using VIA only for women who are less than 50 years old [46].

As demonstrated in the sensitivity analyses, the VIA screening option with a 30% suboptimal sensitivity was not cost-effective compared to the rapid HPV DNA testing options. In the case where VIA is not realistic, rapid HPV DNA testing becomes the most attractive option. The dominance of the rapid HPV DNA testing option over the cytology-based screening option was also reported in China [47]. The use of HPV DNA testing needs to take into consideration its benefits and disadvantages. The advantage of HPV DNA testing is its high sensitivity and specificity, its reproducibility and the fact that the sample can be collected by the patients [46]. However, the test requires appropriate storage and accessibility. Moreover, a positive case does not necessarily mean an abnormal cervix or a cervical cancer, and does not automatically require treatment. The infection mostly regresses spontaneously within 1–2 years and induces acquired immunity with an estimated duration of at least 10 years [48, 49]. Also, a psychological burden has been reported among HPV-positive women [50].

With cytology-based screening, cervical cancer incidence could be reduced by 80% [51] if the sensitivity of cytology and screening coverage are high. LBC has a higher sensitivity than conventional cytology, but its cost is relatively higher [46]. Compared to VIA and rapid HPV DNA testing, a cytology-based screening option is more costly and less effective as a result of lower sensitivity and specificity [17]. Only 55% of true positive cases receive treatment. In this case, the combined VIA and conventional cytology testing option becomes attractive. The use of both tests as primary screening options might improve the detection of precancerous cases in spite of the large number of false positive cases, which leads to a higher rate of colposcopies and biopsies.

Maintaining a high sensitivity implies high programmatic cost both for the VIA and cytology approaches, because of the need of substantial quality control and training [52]. Compared to programmatic cost of cytology, the cost of VIA might consume less resources because it allows large-scale screening. The large-scale screening would reduce the cost of administration and recruitment, which accounts for 25% of the medical direct costs. However, the programmatic cost of VIA might be higher than the cost of rapid HPV DNA testing as the later one requires less resources for quality control and training [52]. Moreover, self-sampling method that could be done with the rapid HPV DNA testing would reduce the cost of screening and increase and maintain the screening coverage [52]. Nonetheless, further studies are desirable to provide more information on this issue.

Decisions regarding a national screening strategy must take into consideration both the advantages and limitations stated above. This could be summarized in terms of affordability, feasibility, accessibility and acceptability. There is therefore a need to further step-up the analyses of these factors in the Lao context prior to making a decision about which option to propose.

### Limitations

Our model has several limitations: it assumes that the natural progression and regression of the cervix state do not depend on the setting. This assumption might under or overestimate progression and regression rates due to the fact that the epidemiological burden of disease is considerably different between countries. Subsequently, this might under or overestimate the cost of precancerous lesion treatment. However, because of the lack of available data on variables such as HPV prevalence, prevalence of CIN and HPV type distribution for lesions and cervical cancers, defining these parameters in the calibration process is difficult. We addressed this problem by conducting probabilistic sensitivity analyses, using the range of values found in the literature.

To simplify model assumptions, we had to assume that only women with high-grade CIN were treated despite the fact that women with low-grade CIN were followed-up every three to six months and were treated if the result remained positive on their second or third test. This might underestimate the cost of screening and treatment as well as the effect of cervical cytology and rapid HPV testing. However, to the best of our knowledge, this should not greatly impact the effectiveness or the total cost because some of the positive low-grade CIN cases will be lost to follow-up, and the cost of treatment of precancerous lesions for positive cases is marginal compared to screening costs.

Our study assumed that all women participated equally in the screening program even in subsequent screenings. This might overestimate screening coverage that could change over time among screened and unscreened women. Screened women with normal test results might not return to the next cycle and vice-versa.

The sensitivities and specificities used in our model were derived from the meta-analysis of available articles worldwide. However, both VIA and cytology-based screening approaches are subjective, and could vary across settings [46]. Future evidence on test performance relevant to the local context might better guide the decision-making processes.

We ignored some costs related to screening and precancerous lesion treatment. These include the cost of specimen delivery and the cost of complications following a treatment. This might underestimate the total cost per person. However, according to Goldhaber-Fiebert [53], these cost components are small relative to the cost of administration and equipment. Therefore, this is unlikely to have a big impact on ICER and should not bias our conclusions. Nevertheless, we based the cost of cancer treatment on Goldie and colleagues [31], which estimated these costs from data coming from other developing countries with similar economic level. Also, we assumed that some invasive cervical cancer cases did not access to treatment. This might underestimate the cost of the baseline scenario. Further investigation on this issue are desirable.

## Conclusions

The combination of vaccination of preadolescent girls and screening was predicted to be more cost-effective than either component alone. Besides VIA, the rapid HPV DNA testing option was predicted to be more cost-effective than a cytology-based screening option or its combination with VIA. Therefore, in addition to girl HPV vaccination program, VIA or rapid HPV DNA should be considered for primary screening of precancerous lesions in Lao PDR.

## Supporting Information

S1 Appendix Detail of methodology and additional results. (DOCX)
