# Supplementary material for: Economic Evaluation of Screening Strategies Combined with HPV Vaccination of Preadolescent Girls for the Prevention of Cervical Cancer in Vientiane, Lao PDR
Source: PLoS One. 2016 Sep 15;11(9):e0162915. doi: 10.1371/journal.pone.0162915 (PMC5025134; doi:10.1371/journal.pone.0162915)
Supplement: S1 Appendix — (DOCX) [file pone.0162915.s001.docx]

**Appendix**

# Methodology

## Simulation overview

Using a mathematical approach, a compartmental dynamic model of the natural history of HPV infection and cervical cancer was constructed and calibrated to reflect the Vientiane capital population in terms of age and sex distribution [[1](#_ENREF_1)], as well as the age-specific incidence and mortality rates related to cervical cancer in 2014 [[2](#_ENREF_2)]. The model consisted of a dynamic cohort population categorized in one-year age groups. The model considered the occurrence of HPV infection and its progression to precancerous lesions and invasive cervical cancer [[3](#_ENREF_3)], according to the probabilities of administrating a context-appropriate treatment for cervical precancerous and invasive cancers. Events defined in the model (such as Cervical Intraepithelial Neoplasia (CIN), cervical cancer, death) were probabilistically monthly imputed to the virtual population over the time course of the simulation. The parameters were retrieved from the literature.

The options included a girl vaccination program and the combination of screening strategies with/without the girl vaccination program. Screening techniques considered included VIA, rapid HPV DNA testing, combined VIA and conventional cytology testing and cytology-based screening.

The virtual population was processed over a period of 100 years. This period of time was used to capture the long-term impact of HPV vaccination [[4](#_ENREF_4)]. Incremental cost/effectiveness ratios were computed on the simulation results. Sensitivity analyses were performed on a specific set of parameters expected to be the most influential on the outcomes [[5](#_ENREF_5)].

## Scenarios

The scenarios consisted of 1) a baseline (no vaccination), 2) a prevention programs consisting of a 10 years old girl HPV vaccination program and/or various pre-cancer screening options. Assumptions on screening strategies were based on feasibility considerations relevant to the Lao context. Currently, according to Lao experts, only cytology, visual inspection with acetic acid (VIA) and rapid HPV DNA testing are available in Vientiane Capital. The following prevention programs were therefore considered:

1. 10 years old girl vaccination
2. 10 years old girl vaccination and VIA screening
3. 10 years old girl vaccination and cytology screening
4. 10 years old girl vaccination and a combined testing of VIA and cytology screening
5. 10 years old girl vaccination and rapid HPV DNA testing
6. VIA screening alone
7. Cytology-based screening alone, either conventional and liquid-based cytology
8. Combination between VIA and cytology screening
9. Rapid HPV DNA testing

In each prevention scenario, screening programs with different initial ages of screening were considered, leading to the following categories of screening target populations:

1. 20-65 years old
2. 25-65 years old
3. 30-65 years old

Moreover, each screening option was evaluated according to the following time-frame, which refers to the current practice in Lao PDR, WHO recommendations and the current practice in some developed countries, respectively.

1. Yearly intervals
2. Three years intervals
3. Five years intervals.

Simulations were performed on various foreseeable combinations of screening options, taking into account the availability of treatments for precancerous lesions in the country (figure 2).

| **Table 1: Precancerous lesions screening options according to initiation age and frequency** | | | | |
| --- | --- | --- | --- | --- |
| **Primary prevention** | **Secondary prevention (screening option)** | **Number of visits** | **Initiation age** | **Frequency** |
| 10 years old girl HPV vaccination | Visual Inspection with acetic acid (VIA) | 1 | 20 years old  25years old  30 years old  (VIA testing ends when women are 45 years old) | Yearly interval  Three years interval  Five years interval |
|  | Cytology testing | 3 |  |  |
|  | Combination between VIA and Cytology testing | 3 |  |  |
|  | Rapid HPV DNA testing | 2 |  |  |

## Model structure

Inspired by previous economic models of HPV vaccination [[6-8](#_ENREF_6)], a dynamic transmission and compartment population-based model was created to reflect the expected effect of HPV vaccination programs, both in females and males. Susceptible girls and boys were considered to be at risk of being infected based on estimated infection rates between partners. For both males and females, the model considered if the HPV genotype was a 16, 18 or other high-risk types, or if it was of low-risk types.

The model considers that among infected women, some lesions regress thanks to a natural immunity against a specific HPV type, but these women remain susceptible to be infected with other HPV types. The infection might also persist and might then progress to Cervical Intraepithelial Neoplasia (low-grade CIN “CIN 1” or high-grade CIN "CIN 2/3”, according to the Richard's modified classification) [[9](#_ENREF_9)]. A low-grade CIN might regress to either immunity state, or infection state [[10-13](#_ENREF_10)] or progress to a high-grade CIN. In case of high-grade CIN, the lesion might regress to immunity state, infection state or low-grade CIN or might progress and become a localized invasive cervical cancer. Localized cancer might progress to regional cancer which might progress then to distant cancer [[14](#_ENREF_14),[15](#_ENREF_15)]. Additionally, women may die of another cause than cervical cancer. Women diagnosed with precancerous lesions will be treated by either Loop Electrosurgical Excision procedure (LEEP) or hysterectomy except in case of VIA screening in which positive cases are treated by cryotherapy. Women with invasive cervical cancer might be symptomatically detected. Diagnosed invasive cervical cancer is treated accordingly, with a defined probability of recovery or treatment failure or death due to treatment complications (figure 1).

In males, the infection might persist or regress conferring them a natural immunity against a defined HPV genotype. The consequences of HPV infection in males, such as warts, were not included in the model because we were only interested in the impact of HPV vaccination on cervical cancer in women. Males could die from general causes (figure 1).

The model assumed that vaccinated people who entered into the vaccine protection compartment remained susceptible for HPV genotypes uncovered by the vaccine; consequently, they had a certain probability of being infected with HPV and getting an invasive cancer. Vaccinated people were susceptible to the 16/18 types HPV infection depending on assumptions done regarding the wane of vaccine immunity (figure 1).

Screening model, a high-grade CIN detected through a cytology-based and rapid HPV DNA testing, led to a treatment with LEEP or a hysterectomy, and to a stage-specific treatment for invasive cervical cancer. In case of VIA screening, a see-and-treat approach was considered, with true positives and false negatives high-grade CIN undergoing a treatment with cryotherapy. Treated cases regressed to healthy state with a specific-type natural immunity. Unscreened or undetected cases or treatment failures follow the natural history of HPV infection and cervical cancer (figure 2).

The model was validated by Lao experts in order to ensure that it realistically reflects the possibilities of routine screening and treating patients in the Vientiane capital context.

## Parameters

The infection rate depended on the age-specific number of new sexual partners per month, the HPV genotype-specific transmissibility and the age-specific HPV prevalence in the opposite sex. To simplify the model, we considered all members of the population as heterosexuals. With each sexual partner, the HPV infection is probabilistically transmitted, depending on genotype-specific transmission probabilities and age-specific HPV prevalence in the opposite-sex population. A sexual relationship matrix group was constructed. The matrix consists of the monthly age-specific probability of having new sexual partners. Each age group has a probability of having a sexual intercourse with someone of the same or a different age group of 0.6 and 0.4 respectively, based on a previous national survey [[16](#_ENREF_16)]. The initial age of sexual intercourse is 15 years old or more in both girls and boys, according to the last survey performed in Vientiane capital city [[17](#_ENREF_17)]. Due to unknown parameters of the number of new sexual partners in Lao PDR, data from the UK [[7](#_ENREF_7)] were used and calibrated to the age-specific incidence of cervical cancer in Lao PDR. The transmissibility of each HPV type was calibrated to take into account the proportion of genotype specific-HPV prevalence and the proportion of cervical cancers due to HPV type 16/18 (see table 3). The proportion of HPV types 16 and 18 among all-type HPV infections was, based on Thai data [[18](#_ENREF_18)], assumed to be 45-50%. These infections may reasonably be assumed to be responsible for approximately 75% of the total incidence of invasive cervical cancer [[19](#_ENREF_19)].

Monthly transition probabilities from one lesion state to another and regression rates were taken from Kim et al [[20](#_ENREF_20)]. For instance, the age-specific monthly probability that a HPV type 16 infection evolves to a low-grade CIN is 0.0047-0.0085, while the rate of transition from low to high-grade CIN is 0.0001-0.0039. The annual rate of detecting an invasive cervical cancer through symptoms is 0.19, 0.6 and 0.9 for local, regional and distant cervical cancers, respectively (see table 4).

In the baseline option, the current conventional cervical cytology screening coverage was fixed at 5.2% every three years [[2](#_ENREF_2)]. The sensitivity and specificity of the cervical cytology and of colposcopy were retrieved from a systematic review and meta-analysis [[21](#_ENREF_21)]. A true positive result of cervical cytology was defined as a high-grade CIN. We assumed that 55% of them would receive the whole treatment regimen, considering 15% loss to follow-up over the three expected visits (for screening, diagnostic test and treatment). The proportion of treatment with LEEP or cryotherapy was based on experts’ opinions. The rate of remission was retrieved from the literature [[22](#_ENREF_22),[23](#_ENREF_23)]. The experts’ panel consisted of two gynecologists with a practice focused on cervical cancer in Lao PDR, Dr. Phongsavan K. and Dr. Marsden E.D.

The proportion of women receiving cancer treatment among diagnosed patients and the stage-specific five-year survival rates due to cancer treatment complications were calibrated based on the estimated mortality rates related to cervical cancer according to Globocan, 2012 (table 4) [[2](#_ENREF_2)].

The sensitivity and specificity of the conventional cervical cytology to detect a high-grade CIN or worse were considered to be 59% (range: 29%-82%) and 94% (range: 88%-99%), respectively [[21](#_ENREF_21)]. Those for liquid-based cervical cytology were 88% (70-94%) and 88% (65-97), respectively [[24](#_ENREF_24)]. Those of VIA were 73.2% (range: 66.5–80%) and 86.7% (range: 82.9–90.4%), respectively [[25](#_ENREF_25)]. Those of the combined VIA and conventional cervical cytology testing were 87% (0.83-90%) and 79% (63-89%), respectively [[26](#_ENREF_26)]. Those of the Rapid HPV DNA testing were 81.5 % (range: 53.1%- 89.5%) and 91.6 % (range: 81.8%-97.4%), respectively [[27](#_ENREF_27)]. The model considers that colposcopy with direct biopsy is used to confirm a positive result from either a cervical cytology test or a rapid HPV DNA testing. The sensitivity and specificity of colposcopy were considered to be 96% (64 –99%) and 48% (30 –93%), respectively. Biopsy was assumed to have a sensitivity and a specificity of 100%. Treatment is provided in two cases: confirmed high-grade CINs and a positive result at the VIA screening test (table 6).

***Precancerous lesions and cancer stage treatment***

The average rate of remission following cryotherapy was considered to be 94% (85-95%) and 86% (83-89%) for low and high-grade CINs, respectively. Success rates for LEEP and hysterectomy were supposed to be 96.7% (90-98%) and 99% (90-100%), respectively [[23](#_ENREF_23)]. The proportion of positive women treated with LEEP or hysterectomy depends on their age. For women aged 35 years or less, it was considered that 80% (50-100%) would be treated with LEEP and 20% (0-50%) with a hysterectomy. For those older than 35 year old, the numbers were reversed: 20% (0-50%) with LEEP and 80% (50-100%) with hysterectomy. The remission rate of stage-specific invasive cervical cancer was calibrated, based on the estimate mortality related to cervical cancer in Lao PDR [[2](#_ENREF_2)] (table 6).

***Compliance***

Patients' compliance was considered at two levels: consent to participate in a screening program and compliance with the health care provider’s recommendations. In all options, base case analyses are performed with a screening coverage assumed to be 50% (range: 10%-80%). Loss to follow-up was assumed to be 15% per visit (range: 0%-50%). Based on a previous study on VIA see-and-treat approach conducted in Lao PDR [[28](#_ENREF_28)], we assumed that all women with a positive screening result accepted to be treated, and that no women underwent a follow-up visit after a precancerous lesion treatment (table 7).

The coverage of HPV vaccination both in girls and boys was assumed to be about 70% (30-80%), with 100% (50-100%) effectiveness against HPV type 16 and 18 and a lifelong protection (10 years to lifelong).

## Model calibration

The population was stratified by gender and age. The model is in the form of a realistic age structured (RAS) model. The equations were numerically solved in Berkeley Madonna version 8.3.18 [[29](#_ENREF_29)]. The model was calibrated using maximum likelihood for the age-specific distribution of the 2014-estimated incidence of cervical cancer and mortality related to cervical cancer data in Lao PDR. Thai data on the prevalence of HPV infection and the prevalence of low-grade and high-grade CIN were used to guide their age-specific distributions. The demographic distribution followed an exponential distribution using UN data to predict the changing birth and death rates over time for Lao PDR [[30](#_ENREF_30)]. To calibrate the age-specific incidence of cervical cancer, we assumed that only the infection rate was different from the Kim et al. model [[20](#_ENREF_20)]. We consequently calculated an infection rate multiplier to calibrate the incidence of cervical cancer according to the Globocan estimates and used under and over estimates in sensitivity analyses (table 5).

The calibration of parameters for the age and stage-specific mortality rates of cervical cancer was conducted by varying the proportion of women receiving treatment for local, regional and distant cancer, the monthly death rates due to treatment complications and the age and stage-specific remission rates. The true proportion of women receiving a treatment in Lao PDR is unknown; we therefore estimated its value according to the experts’ opinion. The best guess of the proportion of women receiving a treatment for a local, regional or distant cancer was 100%, 80% and 70%, respectively.

## Costs

One should stress the fact that no economic evaluation of health interventions has ever been done in Lao PDR. This section refers therefore to a component that required some approximations, as the structure supporting the health care system has not been built to provide the required information for conducting economic evaluations. We recognized that this is a limit, but also considered that undertaking this component would open doors to the realization of further studies on the value of money spent in the Lao PDR health care sector.

The perspective considered was essentially the perspective of the public health care system. Only direct medical costs and the programmatic cost of vaccination implementation were considered.

***Items***

Items were related to the consumption of medical resources for the diagnosis and treatment of cervical cancer and HPV (screening facilities, laboratory, diagnostic tests, hospitalizations, and treatment), as well as the vaccination cost (programmatic cost). A preliminary list of items was built with the help of gynecologists and pathologists working in Lao PDR. These items consisted of:

1. Screening related items: include support items, medical administration, and labor costs. The ingredients of support items consisted of the cost of electricity, water and transportation supplies and other office materials and staffs. Medical administration included training support and medical equipment. Labor cost included the time spent by the gynecologist and the nurse for screening activities. The cytology alone or combined with VIA options requires three visits. The first visit is for screening, the second for receiving the result and making an appointment for positive case. The third is for a colposcopy with direct biopsy. Meanwhile, rapid HPV DNA testing requires two visits. The first is for primary screening, the second for a colposcopy with direct biopsy in case of a positive result. VIA requires only one “see-and-treat approach” visit.
2. Laboratory related items: items were listed according to a pathologist’s advice. Cervical cytology and histology exams included administration, consumable and labor costs. Consumable items for cervical cytology included cover glass, malinol, Gill hemato, OG-6, EA-50, mask, xytene, etanol, and slide. For histology exams, the ingredients included formaline, hematocyline, eosine, paraphine, assette, cyline, obsolute, acetone and malinone. In the Vientiane Capital, four pathology technicians work together and can prepare a total of 50 smear slides for conventional cervical cytology per day. They can also in total prepare 10 histology slides per day. A pathologist needs 20 to 35 minutes for a cytology and histology examination. Other materials used for a cytology examination could not be identified due to lack of information. Meanwhile, the laboratory cost of rapid HPV DNA testing included administration and material costs (table 9).
3. Medication and surgery: the items of precancerous lesions treatment included support activities, drugs, and equipment and labor costs. Cryotherapy is performed in outpatient clinics; LEEP requires one day of hospitalization and simple hysterectomy 7-days.
4. Vaccination included the vaccine cost and programmatic cost, which included micro-planning, training, social mobilization, procurement, logistics, service delivery, supervision and waste management.
5. Programmatic cost of screening included quality control, training, administration and recruitment costs.

***Quantification***

There are no national guidelines for cervical cancer control in Lao PDR. Quantities were therefore estimated based on experts’ opinion.

1. Time spent for screening is supposed to be about 20 minutes for VIA and cervical cytology. Meanwhile, time spent for cervical cytology and histology interpretations is supposed to be about 20 and 35 minutes per case, respectively.
2. The number of visits considered is one for VIA screening and three for other screening strategies.
3. Only consumable items of cytology and histology laboratory were considered. In the Vientiane Capital, four pathology technicians work together and spent a day to prepare 50 to 80 smear lames for conventional and liquid-based cervical cytology, respectively. They also prepare in total 10 histology lames per day. A pathologist needs 20 to 35 minutes per cytology and histology case, respectively.
4. Other quantities were approximated, for instance: hospitalization, surgery

***Item pricing***

Unit prices are reported in the value of 2013 international dollars, using purchasing power parity (PPP). According to WHO, a purchasing power parity (PPP) exchange rate is the number of units of a country currency required to buy the same amounts of goods and services in the domestic market as what can be bought with one U.S. dollar in the United States. International dollars are, therefore, a hypothetical currency allowing comparisons and integration of costs between countries [[31](#_ENREF_31)].

Price per service was calculated by multiplying the cost per unit and the amount of units per service. Unit prices were as often as possible based on data coming from Lao PDR. A Lao hospital unit price list is available. Its numbers have been estimated through a costing survey performed at the departments of gyneco-obstretics of two reference hospitals in the Capital of Vientiane: Mahosot and Setthathirath hospitals in 2013-2014 (personal communication with a head of department of health insurance, Ministry of health, Lao PDR). The survey applied a step down allocation method to estimate the average cost per visit and per hospitalization. Capital costs were not considered due to the difficulty to make an estimation of their real value. Unit prices for missing items were essentially retrieved from the literature. The realism of the valuing procedure was validated by the Lao experts’ committee (table 8).

The price of administration and labor cost in the screening facility are 14.48 I$ and 3.39 I$, respectively. The price of rapid HPV DNA testing is 14.85 I$ per test, based on a previous study performed in rural China [[32](#_ENREF_32)]. We expected this cost to be quite similar to the cost in the Lao context based on assumptions made in the Chinese study for mass screening. We used for the cost of the LEEP the average cost of one-day hospitalization in a gyneco-obstetric ward. The cost of a simple hysterectomy was considered to be the same as the average cost of a surgical operation. The complication of cryotherapy, LEEP and hysterectomy are rare. For that reason, they were not considered (table 8).

The cost of invasive cervical cancer treatment was retrieved from a study done in 72-GAVI eligible countries [[33](#_ENREF_33)]. It includes the costs of treatment for localized, regional and distant cervical cancers (table 10).

***Programmatic cost of screening***

The programmatic costs were based on the literature. The cost of each item was estimated from a proportion of the direct medical cost of vaccination, as calculated in previous studies in developing countries [[32](#_ENREF_32),[34](#_ENREF_34)]. Programmatic cost of VIA screening strategy was estimated at 48% of the total direct medical costs, 23% for quality control and training and 25% for administration and recruitment. The same method of calculation was attributed to a cervical cytology or a combined testing with a VIA program. Programmatic costs for HPV DNA testing was estimated at 35% of the total direct cost, 10% [[32](#_ENREF_32)] and 25% for quality control and training and administration and recruitment, respectively.

***Vaccination cost***

The cost of delivering HPV vaccines consisted of the price of the vaccine and the programmatic cost of vaccination delivery. The programmatic cost of 3-dose HPV vaccine per girl was retrieved from a pilot project on HPV vaccination in 5^th^ grade girls in Vientiane capital in 2014. The vaccine cost per dose was based on the purchasing cost from the Global Alliance for Vaccines and Immunization (GAVI) (4.5 US dollars per dose) [[35](#_ENREF_35)].

## Model Validation process

The model was able to reproduce the 2014 Vientiane Capital expected values regarding demographic data, both for the female and the male populations. However the number of individuals was high for 10 to 25 year old individuals compared to expected values, while it was low for 25-35 year old individuals. The model reproduced results that were consistent with the incidence of cervical cancer and its mortality due to any high-risk HPV type according to the estimates of Globocan 2012. The proportion of cervical cancers related to HPV type 16 and 18 was about 75%. The calibrated infection rate was not different to that reported in the literature (figure 3).

Figure 1: Model structure for natural history of Human Papillomavirus infection and cervical cancer

**Overview of the ordinary differential equations**

**State transition equations**

***Female model (1)***

| ****** |
| --- |

$${S'}_{k,0}=\mu P+\epsilon S_{(k-1),0}+\epsilon S_{(k-1),0}+\omega_{k}{RW}_{k,g,0}-{[\lambda}_{k,g}+{\theta_{k}+(\nu_{k}+\rho}_{k}{)\tau}_{k}+\epsilon]S_{k,0}$$

${S'}_{k,1}={\epsilon S_{(k-1),1}+[(\nu_{k}+\rho}_{k}{)\tau}_{k}]S_{k,0}+ \omega_{k}{RW}_{k,g,1}-{(\lambda}_{k,g}+\varphi+\theta_{k}+\epsilon)S_{k,1}$where vaccinated people remains susceptible for other HPV types rather than type 16/18

$${S'}_{k,2}={\epsilon S}_{(k-1),1}+\varphi S_{k,g,1}+\omega_{k}{RW}_{k,g,2}-{(\lambda}_{k,g}+\theta_{k}+\epsilon){SV}_{k,2}$$

$${I'}_{k,g,0}= \epsilon I_{\left( k-1 \right),g,0}+\lambda_{k,g}[S_{k,0}+(1-{x_{g})RW}_{k,g,0}]+ \alpha_{k,g}{CL}_{k,g,0}+\psi_{k,g}{CH}_{k,g,0}-[\gamma_{k,g}+\eta_{k,g}+\partial_{k,g}{+(\nu_{k}+\rho}_{k}{)\tau}_{k}+\theta_{k}+\epsilon]I_{k,g,0}$$

$${I'}_{k,g,1}= \epsilon I_{\left( k-1 \right),g,1}{+ [(\nu_{k}+\rho}_{k}{)\tau}_{k}]I_{k,g,0}+\lambda_{k,g}[S_{k,1}+(1-{x_{g})RW}_{k,g,1}]+ \alpha_{k,g}{CL}_{k,g,1}+\psi_{k,g}{CH}_{k,g,1}-(\gamma_{k,g}+\eta_{k,g}+\partial_{k,g}+\varphi+\theta_{k}+\epsilon)I_{k,g,1}$$

$${I'}_{k,g,2}= \epsilon I_{\left( k-1 \right),g,2}+\varphi I_{k,g,1} +\lambda_{k,g}[S_{k,2}+(1-{x_{g})RW}_{k,g,2}]+ \alpha_{k,g}{CL}_{k,g,2}+\psi_{k,g}{CH}_{k,g,2}-(\gamma_{k,g}+\eta_{k,g}+\partial_{k,g}+\theta_{k}+\epsilon)I_{k,g,2}$$

$${CL'}_{k,g,0}= {\epsilon{CL}_{\left( k-1 \right),g,0}+\eta_{k,g}I}_{k,g,0}+{\varpi_{k,g}CH}_{k,g,0}+(1-ê_{k})T{CL}_{k,g,0}-[\pi_{k,g}+\alpha_{k,g}+\delta_{k,g}{+(\nu_{k}+\rho}_{k}{)\tau}_{k}+æ_{k}+\theta_{k}+\epsilon]{CL}_{k,g,0}$$

$${CL'}_{k,g,1}= {\epsilon{CL}_{\left( k-1 \right),g,1}{+ [(\nu_{k}+\rho}_{k}{)\tau}_{k}]{CL}_{k,g,0}+\eta_{k,g}I}_{k,g,1}+{\varpi_{k,g}CH}_{k,g,1}+(1-ê_{k})T{CL}_{k,g,1}-(\pi_{k,g}+\alpha_{k,g}+\delta_{k,g}+æ_{k}+\varphi+\theta_{k}+\epsilon){CL}_{k,g,1}$$

$${CL'}_{k,g,2}= {\epsilon{CL}_{\left( k-1 \right),g,2}+\varphi{CL}_{k,g,1}+\eta_{k,g}I}_{k,g,2}+{\varpi_{k,g}CH}_{k,g,2}+(1-ê_{k})T{CL}_{k,g,2}-(\pi_{k,g}+\alpha_{k,g}+\delta_{k,g}+æ_{k}+\theta_{k}+\epsilon){CL}_{k,g,2}$$

$${CH'}_{k,g,0}=\epsilon{CH}_{\left( k-1 \right),g,0}+{\partial_{k,g}I}_{k,g,0}+{\pi_{k,g}CL}_{k,g,0}+{(1-\varepsilon}_{k})T{CH}_{k,g,0}-(\mathcal{F}_{k,g}+\beta_{k,g}+\psi_{k,g}+\varpi_{k,g}{+(\nu_{k}+\rho}_{k}{)\tau}_{k}+\phi_{k}+\theta_{k}+\epsilon){CH}_{k,g,0}$$

$${CH'}_{k,g,1}=\epsilon{CH}_{\left( k-1 \right),g,1}{+ [(\nu_{k}+\rho}_{k}{)\tau}_{k}]{CH}_{k,g,0}+{\partial_{k,g}I}_{k,g,i}+{\pi_{k,g}CL}_{k,g,i}+{(1-\varepsilon}_{k})T{CH}_{k,g,i}-(\mathcal{F}_{k,g}+\beta_{k,g}+\psi_{k,g}+\varpi_{k,g}+\phi_{k}+\varphi+\theta_{k}+\epsilon){CH}_{k,g,i}$$

$${CH'}_{k,g,2}=\epsilon{CH}_{\left( k-1 \right),g,2}+\varphi{CH}_{k,g,1}+{\partial_{k,g}I}_{k,g,2}+2+{(1-\varepsilon}_{k})T{CH}_{k,g,2}-(\mathcal{F}_{k,g}+\beta_{k,g}+\psi_{k,g}+\varpi_{k,g}+\phi_{k}+\theta_{k}+\epsilon){CH}_{k,g,2}$$

$${RW'}_{k,g,0}= \epsilon{RW}_{\left( k-1 \right),g,0}+{\gamma_{k,g}I}_{k,g,i}+\delta_{k,g}{CL}_{k,g,i}+{\beta_{k,g}CH}_{k,g,i}+ \varepsilon_{k}T{CH}_{k,g,i}+ ê_{k}T{CL}_{k,g,0}-[(1-{x_{g})\lambda_{k,g}+\omega_{k}+\theta_{k}{+(\nu_{k}+\rho}_{k}{)\tau}_{k}+\epsilon]RW}_{k,g,0}$$

$${RW'}_{k,g,1}= \epsilon{RW}_{\left( k-1 \right),g,1}{+ [(\nu_{k}+\rho}_{k}{)\tau}_{k}]{RW}_{k,g,0}+{\gamma_{k,g}I}_{k,g,1}+\delta_{k,g}{CL}_{k,g,1}+{\beta_{k,g}CH}_{k,g,1}+ \varepsilon_{k}T{CH}_{k,g,1}+ ê_{k}T{CL}_{k,g,1}-(1-{x_{g})\lambda_{k,g}+\omega_{k}+\theta_{k}+\varphi+\epsilon]RW}_{k,g,1}$$

$${RW'}_{k,g,2}= \epsilon{RW}_{\left( k-1 \right),g,2}+\varphi{RW}_{k,g,1}+{\gamma_{k,g}I}_{k,g,2}+\delta_{k,g}{CL}_{k,g,2}+{\beta_{k,g}CH}_{k,g,2}+ \varepsilon_{k}T{CH}_{k,g,2}+ ê_{k}T{CL}_{k,g,2}-[(1-{x_{g})\lambda_{k,g}+\omega_{k}+\theta_{k}+\epsilon]RW}_{k,g,2}$$

$$\nu_{k}=\frac{\epsilon C_{k}}{1-C_{k}}$$

$$\rho_{k}=\frac{\epsilon{COV}_{k}}{1-{COV}_{k}}$$

***Invasive cervical cancer model (2)***

$${LCC'}_{k,g}=\mathcal{F}_{k,g}{CH}_{k,g}-\left( \left( \mathfrak{D+}\phi_{k} \right)\mathcal{g}+h+\theta_{k}+\epsilon\right){LCC}_{k,g}$$

$${RCC'}_{k}=h{LCC}_{k}-\left( \left( \mathbb{Q+}\phi_{k} \right)\Omega\mathcal{+H+}\theta_{k}+\epsilon\right){RCC}_{k}$$

$${DCC'}_{k}\mathcal{=H}{RCC}_{k}-\left( \left( \mathcal{L+}\phi_{k} \right)\mathcal{A+}⋏_{k}+\theta_{k}+\epsilon\right){DCC}_{k}$$

$${LCCd'}_{k,g}=\left( \psi+\phi_{k} \right){\mathcal{g}LCC}_{k,g}-\left( h+\mathcal{m}_{k}+\theta_{k}+\epsilon\right){LCCd}_{k,g}$$

$${RCCd'}_{k}=\left( \mathbb{Q+}\phi_{k} \right){\Omega RCC}_{k}+(1-\mathcal{r}_{k})h{LCCd}_{k}-\left( \mathcal{H+}\mathcal{n}_{k}+\theta_{k}+\epsilon\right){RCCd}_{k}$$

$${DCCd'}_{k}=\left( \mathcal{L+}\phi_{k} \right){\mathcal{A}DCC}_{k}+(1-\mathfrak{y}_{k}){\mathcal{H}RCCd}_{k}-\left( \ni_{k}+\theta_{k}+\epsilon\right){DCCd}_{k}$$

$${RC'}_{k}=\mathcal{r}_{k}h{LCCd}_{k}+\mathfrak{y}_{k}{\mathcal{H}RCCd}_{k}-\left( \theta_{k}+\epsilon\right){RC}_{k}$$

***Male model (3)***

| 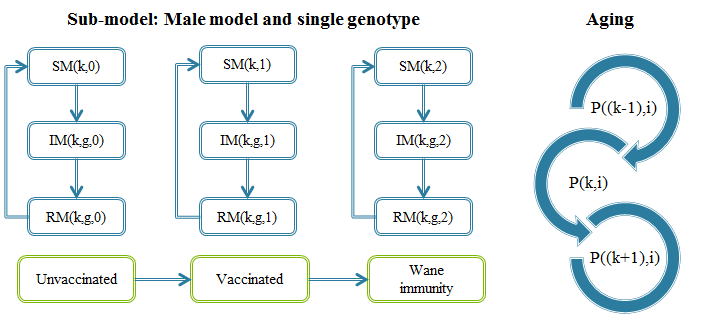 |
| --- |

$${SM'}_{k,0}=\mu\mathcal{o}P+\epsilon{SM}_{(k-1),0}+\omega_{k}{RM}_{k,g,0}-{[\lambda}_{k,g}+{\theta_{k}+(\nu_{k}+\rho}_{k}{)\tau}_{k}+\epsilon]{SM}_{k,0}$$

${SM'}_{k,1}=\epsilon{SM}_{(k-1),1}+[{(\nu_{k}+\rho}_{k}{)\tau}_{k}]{SM}_{k,0} {{+ \omega}_{k}RM}_{k,g,1}- {(\lambda}_{k,g}+\theta_{k}+\varphi+\epsilon){SM}_{k,1}$ where vaccinated people remains susceptible for other HPV types rather than type 16/18

$${SM'}_{k,2}=\epsilon{SM}_{(k-1),2}+\varphi{SM}_{k,1}+{\omega_{k}RM}_{k,g,2}-{(\lambda}_{k,g}+\theta_{k}+\epsilon){SM}_{k,2}$$

$${IM'}_{k,g,0}= \epsilon{RM}_{\left( k-1 \right),g,0}+\lambda_{k,g}[{SM}_{k,0}+(1-{x_{g})RM}_{k,g,0}]-[\gamma_{k,g}{+(\nu_{k}+\rho}_{k}{)\tau}_{k}+\theta_{k}+\epsilon]{IM}_{k,g,0}$$

$${IM'}_{k,g,1}= \epsilon{RM}_{\left( k-1 \right),g,1}+[{(\nu_{k}+\rho}_{k}{)\tau}_{k}]{IM}_{k,g,0}+\lambda_{k,g}[{SM}_{k,1}+\left( 1-{x_{g})RM}_{k,g,1} \right]-[\gamma_{k,g}+\varphi+\theta_{k}+\epsilon]{IM}_{k,g,1}$$

$${IM'}_{k,g,2}=\epsilon{RM}_{\left( k-1 \right),g,2}+\varphi{IM}_{k,g,1}+ \lambda_{k,g}[{SM}_{k,2}+\left( 1-{x_{g})RM}_{k,g,2} \right]-[\gamma_{k,g}-\left( \gamma_{k,g}+\theta_{k}+\epsilon\right){]IM}_{k,g,2}$$

$${RM'}_{k,g,0}=\epsilon{RM}_{\left( k-1 \right),g,0}+ {\gamma_{k,g}IM}_{k,g,0}-[(1-{x_{g})\lambda_{k,g}+\omega_{k}+{\theta_{k}+(\nu_{k}+\rho}_{k}{)\tau}_{k}+\epsilon]RM}_{k,g,0}$$

${RM'}_{k,g,1}=\epsilon{RM}_{\left( k-1 \right),g,1}+{\gamma_{k,g}IM}_{k,g,1}+[{(\nu_{k}+\rho}_{k}{)\tau}_{k}]{RM}_{k,0}-( (1-{x_{g})\lambda_{k,g}+\omega_{k}+\varphi+\theta_{k}+\epsilon)RM}_{k,g,1}$where vaccinated people remains susceptible for other HPV types rather than type 16/18

$${RM'}_{k,g,2}=\epsilon{RM}_{\left( k-1 \right),g,2}+\varphi{RM}_{k,g,1}+{\gamma_{k,g}IM}_{k,g,2}-[(1-{x_{g})\lambda_{k,g}+\omega_{k}+\theta_{k}+\epsilon]RM}_{k,g,2}$$

***Precancerous lesions treatment model (4)***

$${TCL'}_{k,g,0}= {\epsilon{TCL}_{\left( k-1 \right),g,0}+æ_{k}CL}_{k,g,i} -[(\theta_{k}+{(1-\varepsilon}_{k})+\varepsilon_{k}{+(\nu_{k}+\rho}_{k}{)\tau}_{k}+\epsilon]T{CL}_{k,g,0}$$

$${TCL'}_{k,g,1}= {\epsilon{TCL}_{\left( k-1 \right),g,1}+æ_{k}CL}_{k,g,i} +[{(\nu_{k}+\rho}_{k}{)\tau}_{k}]{TCL}_{k,g,0}-[(\theta_{k}+{(1-\varepsilon}_{k})+\varepsilon_{k}+\varphi+\epsilon]T{CL}_{k,g,1}$$

$${TCL'}_{k,g,2}= {{\epsilon{TCL}_{\left( k-1 \right),g,2}+\varphi{TCL}_{k,g,1}+æ}_{k}CL}_{k,g,i} -[(\theta_{k}+{(1-\varepsilon}_{k})+\varepsilon_{k}+\epsilon]T{CL}_{k,g,2}$$

$${TCH'}_{k,g,0}= {\epsilon{TCH}_{\left( k-1 \right),g,0}+\phi_{k}CH}_{k,g,0} -[(\theta_{k}+{(1-\varepsilon}_{k})+\varepsilon_{k}{+(\nu_{k}+\rho}_{k}{)\tau}_{k}+\epsilon]T{CH}_{k,g,0}$$

$${TCH'}_{k,g,1}= {{\epsilon{TCH}_{\left( k-1 \right),g,1}+\phi}_{k}CH}_{k,g,1} -[(\theta_{k}+{(1-\varepsilon}_{k})+\varepsilon_{k}+\varphi+\epsilon]T{CH}_{k,g,1}$$

$${TCH'}_{k,g,2}= {\epsilon{TCH}_{\left( k-1 \right),g,2}+\phi_{k}CH}_{k,g,2} -[(\theta_{k}+{(1-\varepsilon}_{k})+\varepsilon_{k}+\epsilon]T{CH}_{k,g,2}$$

$$\phi_{k}= \varsigma_{k}\varrho(1-los\_final)$$

***Force of infection (5)***

$$\lambda_{k,g}=A\sum_{k=1}^{N} {Lamda}_{k,g}$$

Where A is the adjustment of the total estimated force of infection, and N is the total number of age group and

${Lamda}_{k,g}= \frac{\mathcal{T}_{g}M_{k}{IM}_{k,g}}{{POPM}_{k}}$ for female

$${POPM}_{k}={STT}_{k}+{IM}_{k}+RM+{VM}_{k}$$

And

${Lamda}_{k,g}= \frac{\mathcal{T}_{g}M_{k}{(IM}_{k,g}+{CL}_{k,g}+{CH}_{k,g})}{{POPF}_{k}}$ for male

$${POPF}_{k}=S_{k}+I_{k}+CL+{CH}_{k}+RW+{LCC}_{k}+{RCC}_{k}+{DCCd}_{k}+{LCCd}_{k}+{RCCd}_{k}+{DCCd}_{k}$$

Where M is the contact matrix

$M_{k}= {\mathfrak{s}_{1}MS}_{k}+\frac{{\mathfrak{s}_{2}MS}_{k}}{NG-1}$ where MS is new sexual partnership per month; $\mathfrak{s}_{1}$is the probability of having a sexual partner within the same age group; $\mathfrak{s}_{2}$is the probability of having a sexual partner within a different age group; and NG is the total number of age

***Calibration: maximum likelihood estimation (6)***

${LI}_{k,g}= {ICD}_{k,g}\ln\left( IC \right)-IC$ where ICD is the observed incidence of invasive cervical cancer, and IC is expected incidence of invasive cervical cancer

| **Table 1: Abbreviation of the model structure variables** | |
| --- | --- |
| **Variable** | **Meaning** |
| $S_{k,..}$ | Healthy women (age k, 0 is unvaccinated, 1 is vaccinated and 2 is waned status) at time t |
| $I_{k,g,..}$ | Infection in females (age k, genotype g, 0 is unvaccinated, 1 is vaccinated and 2 is waned status) at time t |
| ${RW}_{k,g,..}$ | Regression of infection or precancerous lesions (age k, genotype g, 0 is unvaccinated, 1 is vaccinated and 2 is waned status) at time t |
| ${CL}_{k,g,..}$ | Low-grade Cervical Intraepithelial Neoplasia (age k, genotype g, 0 is unvaccinated, 1 is vaccinated and 2 is waned status) at time t |
| ${CH}_{k,g,..}$ | High-grade Cervical Intraepithelial Neoplasia (age k, genotype g, 0 is unvaccinated, 1 is vaccinated and 2 is waned status) at time t |
| ${LCC}_{k,g}$ | Undetected local cancer (age k, genotype g) at time t |
| ${RCC}_{k}$ | Undetected regional cancer (age k) at time t |
| ${DCC}_{k}$ | Undetected distant cancer (age k) at time t |
| ${LCCd}_{k,g}$ | Detected local cancer (age k, genotype g) at time t |
| ${RCCd}_{k}$ | Detected regional cancer (age k) at time t |
| ${DCCd}_{k}$ | Detected distant cancer (age k) at time t |
| ${RC}_{k}$ | Recovery from cancer treatment (age k) at time t |
| *P* | Total female population |
| ${SM}_{k,..}$ | Healthy males (age k, 0 is unvaccinated, 1 is vaccinated and 2 is waned status) at time t |
| ${IM}_{k,g,..}$ | Infection in males (age k, genotype g, 0 is unvaccinated, 1 is vaccinated and 2 is waned status) at time t |
| ${RM}_{k,g,..}$ | Recovery with natural immunity in males (age k, genotype g, 0 is unvaccinated, 1 is vaccinated and 2 is waned status) at time t |
| ${TCL}_{k,g,..}$ | Women with low-grade CIN receiving treatment (age k, genotype g, 0 is unvaccinated, 1 is vaccinated and 2 is waned status) at time t |
| ${TCH}_{k,g,..}$ | Women with high-grade CIN receiving treatment (age k, genotype g, 0 is unvaccinated, 1 is vaccinated and 2 is waned status) at time t |
| ${POPF}_{k}$ | Total female population (age k) |
| ${POPM}_{k}$ | Total male population (age k) |
| IW16 | HPV type 16 infected women |
| IW18 | HPV type 18 infected women |
| IW_H | Other high-risk HPV infected women |
| RW16 | Clearing up HPV type 16 infection with natural immunity against HPV type 16 |
| RW18 | Clearing up HPV type 18 infection with natural immunity against HPV type 18 |
| RW_H | Clearing up other high-risk HPV infection with natural immunity against high-risk HPV |
| DG | Death due to other causes |
| DC | Death due to cervical cancer |

| **Table 2: Abbreviation of model structure parameters** | |
| --- | --- |
| **Parameters** | **Meaning** |
| $\epsilon$ | Aging rate |
| $\mu$ | Birth rate |
| $\omega_{k}$ | Waning of HPV natural immunity (age k) |
| $\psi_{k,g}$ | Waning of HPV vaccine-induced immunity (age k, genotype g) |
| $\gamma_{k,g}$ | Regression rate from infection to healthy state (age k, genotype g) |
| $\delta_{k,g}$ | Regression rate from low-grade CIN to healthy state (age k, genotype g) |
| $\alpha_{k,g}$ | Regression rate from low-grade CIN to infection (age k, genotype g) |
| $\beta_{k,g}$ | Regression rate from high-grade CIN to healthy state (age k, genotype g) |
| $\varepsilon_{k}$ | Cure rate of high-grade Cervical Intraepithelial Neoplasia treatment (age k) |
| $ê_{k}$ | Cure rate of low-grade Cervical Intraepithelial Neoplasia treatment (age k) |
| $\nu_{k}$ | Preadolescent vaccination coverage (age k) |
| $\lambda_{k,g}$ | Infection rate (age k, genotype g) |
| $\theta_{k}$ | Death rate due to other causes in women (age k) |
| $\tau_{k}$ | Effectiveness of the vaccine (age k) |
| $\rho_{k}$ | Vaccination coverage for catch-up component (age k) |
| $\eta_{k,g}$ | Progression rate from infection to low-grade CIN (age k, genotype g) |
| $\partial_{k,g}$ | Progression rate from infection to high-grade CIN (age k, genotype g) |
| $x_{g}$ | Effectiveness of the natural immunity (age k) |
| $\pi_{k,g}$ | Progression rate from low-grade CIN to high-grade CIN (age k, genotype g) |
| $\mathcal{F}_{k,g}$ | Progression rate from high-grade CIN to invasive cervical cancer (age k, genotype g) |
| $h$ | Progression rate from local cervical cancer to regional cervical cancer |
| $\mathcal{H}$ | Progression rate from regional cervical cancer to distant cervical cancer |
| $\psi_{k,g}$ | Regression rate from high-grade CIN to infection (age k, genotype g) |
| $\varpi_{k,g}$ | Regression rate from high-grade CIN to low-grade CIN (age k, genotype g) |
| $\mathfrak{D}$ | Symptomatic detection rate of local cervical cancer |
| $\mathbb{Q}$ | Symptomatic detection rate of regional cervical cancer |
| $\mathcal{L}$ | Symptomatic detection rate of distant cervical cancer |
| $\phi_{k}$ | Detection rate through screening for high-grade CIN (age k) |
| $æ_{k}$ | Detection rate through screening for low-grade CIN (age k) |
| $\mathcal{r}_{k}$ | Cure rate of local cervical cancer (age k) |
| $\mathfrak{y}_{k}$ | Cure rate of regional cervical cancer (age k) |
| $⋏_{k}$ | Death rate due to distant cervical cancer in women who do not receive treatment (age k) |
| $\ni_{k}$ | Death rate due to distant cervical cancer in women who receive treatment (age k) |
| $\mathcal{m}_{k}$ | Death rate due to local cervical cancer treatment (age k) |
| $\mathcal{n}_{k}$ | Death rate due to regional cervical cancer treatment (age k) |
| $los\_final$ | Proportion of loss to follow-up at three visits |
| $C$ | Proportion of vaccinated preadolescent girls/boy vaccination |
| $COV$ | Proportion of people given a catch-up component |
| $\varsigma_{k}$ | Screening coverage at age class k (age k) |
| $\varrho$ | Sensitivity of screening test |
| $\mathcal{g}$ | Proportion of women with local cervical cancer who accept the treatment |
| $\Omega$ | Proportion of women with regional cervical cancer who accept the treatment |
| $\mathcal{A}$ | Proportion of women with distant cervical cancer who accept the treatment |
| $\mathcal{o}$ | Male to female population ratio |
| $\mathcal{T}_{g}$ | Genotype-specific transmission probability |

Figure 2: The screening model for cytology, VIA, rapid HPV DNA testing and combined testing VIA and cytology


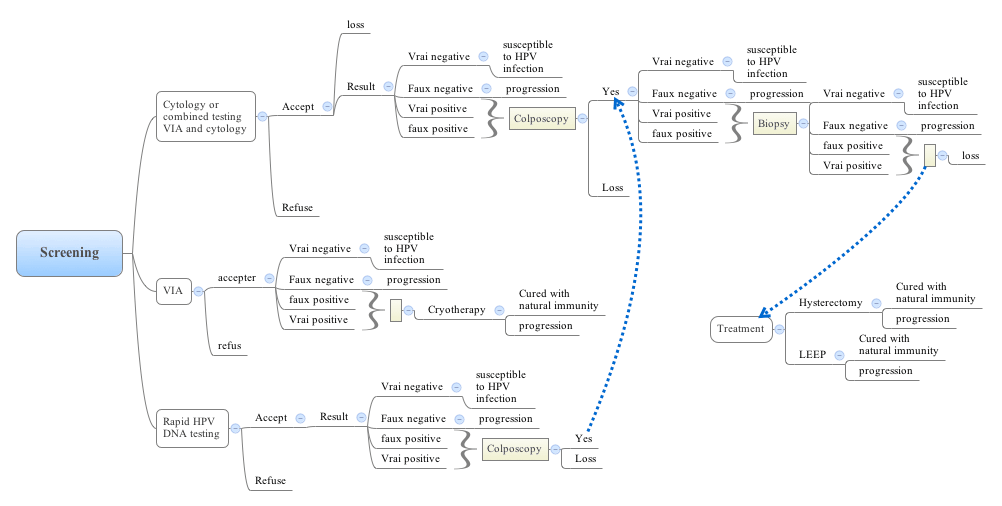


| **Note:** The cytology alone option or combined with VIA is a three-visit approach. The first visit refers to a primary screening; second refers to receiving the result and making an appointment for positive case. Third refers to colposcopy with direct biopsy. Meanwhile, rapid HPV DNA testing is a two-visit approach. The first visit refers to primary screening. Second refers to colposcopy with direct biopsy in positive case. VIA is considered as single-visit approach “see-and-treat approach” |
| --- |

**Figure 3: Model calibration to age-specific incidence and mortality of cervical cancer**

| **Table 3: Model parameters: force of infection** | | | | | |
| --- | --- | --- | --- | --- | --- |
| **Age group** | **Male** | **Female** | **Adjusted ¶** | **Multiplier ‡** | **Source** |
| **Transmissibility per sexual partnership** | | | | | Calibrated |
| HPP 16 | 0.355 | 0.355 |  |  |  |
| HPV 18 | 0.40 | 0.40 |  |  |  |
| Other-HR HPV | 0.41 | 0.41 |  |  |  |
| Low-risk HPV | 0.39 | 0.39 |  |  |  |
| **Mean number of annual change of sexual partners among males and females** | | | | | [[36](#_ENREF_36)] |
| 12-13 | 0.222 | 0.071 | 1 | 2.48-4.43 |  |
| 14-15 | 0.673 | 0.283 | 1 |  |  |
| 15-19 | 3.794 | 2.48 | 0.7 |  |  |
| 20-24 | 5.802 | 2.442 | 0.7 |  |  |
| 25-29 | 2.957 | 1.728 | 0.7 |  |  |
| 30-34 | 2.113 | 0.971 | 0.7 |  |  |
| 35-39 | 1.323 | 0.842 | 0.7 |  |  |
| 40-44 | 1.323 | 0.842 | 1 |  |  |
| 45-49 | 0.662 | 0.421 | 1 |  |  |
| 50-54 | 0.662 | 0.421 | 2 |  |  |
| 55-64 | 0.331 | 0.211 | 2 |  |  |
| 65-74 | 0.166 | 0.106 | 3 |  |  |
| **Sexual mixing matrix** | | | | | [[16](#_ENREF_16)] |
| Same age | 0.6 | 0.6 |  |  |  |
| Different age | 0.4 | 0.4 |  |  |  |
| **¶** Adjusted values was applied to the force of infection model  **‡** Multiplier values ranged according to related-scenarios of annual incidence rate of cervical cancer | | | | | |

| **Table 4: Summary of input parameters for the model** | | | | |
| --- | --- | --- | --- | --- |
| **Parameters** | | | **Baseline values*** | **Source** |
| **Progression** | | | | |
| Healthy to infection † (-20 and +40%) | HPV-16 | | 0.000175-0.003148 (0.0001426-0.00761) | Calibrated |
|  | HPV-18 | | 0.0004-0.000789 (0.000102-0.00168) |  |
|  | Other HR HPV | | 0.000206-0.004038 (0.0001703-0.00911) |  |
|  | LR HPV | | 0.000958-0.018412 (0.00069-0.0537) |  |
| HPV DNA to CIN1‡ | HR-16 HPV | | 0.005194-0.00901 | [[20](#_ENREF_20)] |
|  | HR-18 HPV | | 0.002793-0.004845 |  |
|  | HR-other HPV | | 0.007693-0.013345 |  |
|  | LR-HPV | | 0.002397-0.001222 |  |
| Proportion (%) of women who transition directly from HPV DNA to CIN2,3 | HR-16 HPV | | 0.64 |  |
|  | HR-18 HPV | | 0.975 |  |
|  | HR-other HPV | | 0.966 |  |
|  | LR-HPV | | 0.98 |  |
| CIN 1 to CIN 2,3 ‡ | HR-16 HPV | | 0.00951-0.012363 |  |
|  | HR-18 HPV | | 0.0051-0.00663 |  |
|  | HR-other HPV | | 0.00747-0.009711 |  |
|  | LR-HPV | | 0.000149-0.000222 |  |
| CIN 2,3 to local cancer | HR-16 HPV | | 0.000151-0.00906 |  |
|  | HR-18 HPV | | 0.000264-0.01584 |  |
|  | HR-other HPV | | 0.000199-0.01194 |  |
| Local to regional invasive cancer | | | 0.0200 |  |
| Regional to distant invasive cancer | | | 0.0250 |  |
| **Regression** | | | |  |
| HPV DNA to Normal | HR-16 HPV | 0.09089 | |  |
|  | HR-18 HPV | 0.09089 | |  |
|  | HR-other HPV | 0.09272 | |  |
|  | LR-HPV | 0.09699 | |  |
| CIN 1 to normal ‡‡ | HR-16 HPV | 0.03782 | |  |
|  | HR-18 HPV | 0.03782 | |  |
|  | HR-other HPV | 0.04575 | |  |
|  | LR-HPV | 0.01708 | |  |
| CIN 2,3 to Normal §§ | HR-16 HPV | 0.000798-0.000455 | |  |
|  | HR-18 HPV | 0.003556-0.011938 | |  |
|  | HR-other HPV | 0.002926-0.009823 | |  |
|  | LR-HPV | 0.001904-0.006392 | |  |
| Other | | | |  |
| Immunity (%) (HR-HPV types only) ¶¶ | HR-16 HPV | 0.66 | |  |
|  | HR-18 HPV | 0.86 | |  |
|  | HR-other HPV | 0.59 | |  |
| Annual probability of symptom detection # | Local invasive cancer | 0.33 | |  |
|  | Regional invasive cancer | 0.60 | |  |
|  | Distant cancer | 0.9 | |  |
| Proportion of cancer patient receiving the treatment | Local cancer | 100% | | Assumption |
|  | Regional cancer | 87% | |  |
|  | Distant cancer | 78% | |  |
| Age-specific 5-year survival proportion after diagnosis and treatment (%) £ | Local cancer | 0.29-71% | | Calibrated |
|  | Regional cancer | 0.24-78% | |  |
| Age-specific monthly probability of death | Complication of local cancer treatment | 0.012-0.037 | | Calibrated |
|  | Complication of regional cancer treatment | 0.0098-0.028 | |  |
|  | Distant cancer (rate) | 0.28-0.83 | |  |
| Age-specific all cause death rates per person per year | Female | 0,00106-0,4122 | | [[37](#_ENREF_37)] |
|  | Male | 0.001-0.47 | |  |
| * Baseline values are monthly age-specific probabilities, unless otherwise noted  † The transition from healthy state to infection is a force of infection derived from the number of sexual partner change, HPV type-specific transmissibility.  ‡ HPV, human papillomavirus; CIN, cervical intraepithelial neoplasia; HR, high risk; LR, low risk  ‡‡ 70% of women with CIN 1 regress to normal, 30% to HPV.  §§ 70% of women with CIN2,3 regress to normal, 15% to HPV, 15% to CIN 1.  ¶¶ Immunity represents the degree to protection each woman faces against future type-specific infection after infection after first infection and clearance. The immunity was assumed to be lifelong.  # The annual probability of symptom detection corresponds to 15% for local cancer and 85% for advanced cancer  £ Age-specific survival proportion was calibrate, based on a mortality rate estimated by Globocan [[2](#_ENREF_2)]. | | | | |

| **Table 5: Calibration target** | | | | | |
| --- | --- | --- | --- | --- | --- |
| **Calibration target** | | **Source** | **Calibration target** | | **Source** |
| **Female population¶** | | [[1](#_ENREF_1)] | **Annual incidence rates of invasive cervical cancer per 100,000** | | [[2](#_ENREF_2)] |
| 0- <5 | 44196 |  | 15-39 | 5.2 |  |
| 5- <10 | 40488 |  | 40-44 | 26.9 |  |
| 10 - <15 | 27947 |  | 45-49 | 33.3 |  |
| 15 - < 20 | 31402 |  | 50-54 | 37.1 |  |
| 20 - < 25 | 38205 |  | 55-59 | 36.9 |  |
| 25 - < 30 | 48941 |  | 60-64 | 34.8 |  |
| 30 - < 35 | 45627 |  | 65-69 | 33.7 |  |
| 35 - < 40 | 32125 |  | 70-74 | 30.5 |  |
| 40 - < 45 | 26762 |  | >74 | 29 |  |
| 45 - < 50 | 21895 |  | **Annual mortality of invasive cervical cancer per 100,000** | |  |
| 50 - < 55 | 17307 |  | 15-39 | 1.2 |  |
| 55 - < 60 | 12766 |  | 40-44 | 9.6 |  |
| 60 - < 65 | 8251 |  | 45-49 | 14.2 |  |
| 65 - < 70 | 5,930 |  | 50-54 | 19.8 |  |
| 70 - < 75 | 4152 |  | 55-59 | 23.9 |  |
| 75+ | 6119 |  | 60-64 | 27.9 |  |
| **Distribution of HPV types among women with cancer, Thai data** | | [[18](#_ENREF_18)] | 65-69  70-74  >74 | 31.8  35.6  39.4 |  |
| HPV1618 | 75.1 |  |  |  |  |
| Other-HR HPV‡ | 24.9 |  |  |  |  |
| ¶ The proportion of male population to female population is 0.948  ‡ HPV, human papillomavirus | | | | | |

Table 6: Summary of input other parameters for the model

| **Parameters** | **Value (range)** | **Distribution** | **Source** |
| --- | --- | --- | --- |
| **VIA** |  |  |  |
| Sensitivity (95% Confidence interval) | 73.2% (66.5–80.0%) | Beta | [[25](#_ENREF_25)] |
| Specificity (95% CI) | 86.7% (82.9–90.4%) | Beta |  |
| **Conventional cervical cytology** |  |  |  |
| Sensitivity for CIN23 | 59% (29-82%) | Beta | [[21](#_ENREF_21)] |
| Specificity | 94% (88-99%) | Beta |  |
| **ThinPrep Cervical cytology** |  |  |  |
| Sensitivity for CIN23 | 88% (70-94%) | Beta | [[21](#_ENREF_21),[38](#_ENREF_38)] |
| Specificity | 88% (65-97%) | Beta | [[24](#_ENREF_24)] |
| **Combined testing VIA and conventional cytology** |  |  |  |
| Sensitivity to detect high-grade CIN | 87% (83-90%) | Beta | [[26](#_ENREF_26)] |
| Specificity | 79% (63-89%) | Beta |  |
| **Rapid HPV DNA testing** |  |  |  |
| Sensitivity to detect high-grade CIN | 81.5 % (76.5- 85.8%) | Beta | [[27](#_ENREF_27)] |
| Specificity | 91.6 % (81.8%-97.4%) | Beta |  |
| **Colposcopy** |  |  | [[39](#_ENREF_39)] |
| Sensitivity for high-grade CIN | 96% (64 –99%) | Beta |  |
| Sensitivity | 48% (30 –93%) | Beta |  |
| **Probability of treatment for High grade CIN in Cervical cytology** |  |  |  |
| ≤ 35 years | LEEP: 80% (50-80%)  Hysterectomy: 20% (20-50%) | Beta | Assumption # |
| > 35 years | Hysterectomy: 80% (50-80%)  LEEP: 20% (20-50%) | Beta |  |
| **Proportion of recovery** |  |  |  |
| Cryotherapy |  |  |  |
| Low-grade CIN | 94% (85-95) | Beta | [[40](#_ENREF_40)] |
| High-grade CIN | 86% (83-89) | Beta |  |
| LEEP: High-grade CIN | 96.7% (90-98 %) | Beta | [[22](#_ENREF_22)] |
| Hysterectomy: Any CIN | 99% (90-100%) | Beta | [[41](#_ENREF_41)] |
| Local cervical cancer |  | Beta | Calibrated |
| Regional cervical cancer |  | Beta | Calibrated |
| Mortality related to invasive cancer treatment |  | Beta | Calibrated |
| Local cervical cancer |  | Beta | Calibrated |
| Regional cervical cancer |  |  | Calibrated |
| Age-specific mortality of all-cause mortality |  |  |  |
| Vaccine efficacy against HPV type 16 and 18 infection | 100% |  | [[42](#_ENREF_42)] |
| **Note:**  # Assumption was based on experts’ opinion  Women with local cervical cancer are treated by hysterectomy  Women with regional cervical cancer are treated by chemoradiation  Women with distant cancer are given palliative care | | | |

**Table 7: compliance**

| **Item** |  | **Percentage (%)** | **Distribution** |
| --- | --- | --- | --- |
| Screening coverage (assumptions according to experts) | No screening program (VIA and cervical cytology) | 5% (0-20%) | Beta |
|  | Screening program (Cervical cytology or VIA or Rapid HPV DNA testing) | First time: 70% (30-70%) | Beta |
|  |  | Following time: 10% (0-50) | Beta |
| Loss of follow-up of at screening visit, according to statistics at the pathology center | Per visit | 15% (0-50) | Beta |
| Among women with suspicion of invasive cervical cancer, percentage undergoing a full diagnosis procedure |  | 60% (40-100) | Beta |
| Percentage of women with local cervical cancer undergoing treatment | Surgery | 80% (50-100) | Beta |
|  | Other (palliative or nor care) | 20% (0-50) | Beta |
|  | Loss to follow-up | 10% (0-50) | Beta |
| Percentage of women with regional cervical cancer undergoing a treatment in Thailand/Vietnam/China | Chemoradiation | 80% (0-50) | Beta |
|  | Loss to follow-up | 20% (0-50) | Beta |
| Percentage of women with distant cervical cancer receiving palliative care | No care | 80% | Beta |

**Table 8: Costing parameters**

| **Option** | **Items** | **Unit price (2013 I$)** | **Source** |
| --- | --- | --- | --- |
| VIA | Administration ‡ | 14.48 | Personal communication with a head of department of health insurance. Ministry of health, Lao PDR |
|  | Medical staff ¶ | 3.39 |  |
|  | **Subtotal** | 17.87 |  |
|  | Programmatic cost § | 8.58 | [[32](#_ENREF_32),[34](#_ENREF_34)] |
|  | **Total** | 26.45 |  |
| Conventional cervical cytology | Administration ‡ | 14.48 | Personal communication with a head of department of health insurance. Ministry of health, Lao PDR |
|  | Medical staffs ¶ | 3.39 |  |
|  | Cervical cytology laboratory equipment | 11.20 | Personal communication with a head of department of Pathology center, University of Health Science, Ministry of Health, Lao PDR |
|  | Laboratory staffs | 3.54 |  |
|  | **Subtotal** | 32.61 |  |
|  | Programmatic cost § | 15.65 | [[32](#_ENREF_32),[34](#_ENREF_34)] |
|  | **Total** | 48.27 |  |
| Liquid-based (Thin-Prep) cervical cytology | Administration ‡ | 14.48 | Personal communication with a head of department of health insurance. Ministry of health, Lao PDR |
|  | Medical stuff ¶ | 3.39 |  |
|  | Cervical cytology laboratory equipment | 20.96 | Personal communication with a head of department of Pathology center, University of Health Science, Ministry of Health, Lao PDR |
|  | Laboratory staffs | 4.55 |  |
|  | **Subtotal** | 43.39 |  |
|  | Programmatic cost § | 20.83 | [[32](#_ENREF_32),[34](#_ENREF_34)] |
|  | **Total** | 64.21 |  |
| VIA+ Conventional cervical cytology | Administration ‡ | 14.48 | Personal communication with a head of department of health insurance. Ministry of health, Lao PDR |
|  | Medical stuff ¶ | 6.78 |  |
|  | Cervical cytology laboratory equipment | 11.20 | Personal communication with a head of department of Pathology center, University of Health Science, Ministry of Health, Lao PDR |
|  | Laboratory staffs | 3.56 |  |
|  | **Subtotal** | 36.03 |  |
|  | Programmatic cost § | 14.89 | [[32](#_ENREF_32),[34](#_ENREF_34)] |
|  | **Total** | 50.91 |  |
| Rapid test of HPV DNA testing | Administration ‡ | 14.48 | Personal communication with a head of department of health insurance. Ministry of health, Lao PDR |
|  | Medical stuff ¶ | 3.39 |  |
|  | Cervical cytology laboratory equipment | 14.85 | Personal communication with a head of department of Pathology center, University of Health Science, Ministry of Health, Lao PDR |
|  | Laboratory staffs | 2.23 |  |
|  | **Subtotal** | 34.94 |  |
|  | Programmatic cost § | 12.23 | [[32](#_ENREF_32),[34](#_ENREF_34)] |
|  | **Total** | 47.18 |  |
| Colposcopy | Administration ‡ | 14.48 | Personal communication with a head of department of health insurance. Ministry of health, Lao PDR |
|  | Medical staff ¶ | 3.39 |  |
|  | **Total** | 17.87 |  |
| ‡ Administration includes general and medical administration. General administration includes electricity, water and transportation supplies and other office martials and stuffs. Medical administration included training support and aids, and some medical equipment.  ¶ Monthly salary also includes incentives, gasoline and overtime pay. Salary per hour = salary per day/8; Salary per day =(monthly salary x 12 months) / (52 weeks x 5 working days).   - Monthly average salary of gynecologist is 1303 dollars - Monthly average salary of nurse is 736 dollars - Monthly average salary of pathologist is 992 dollars - Monthly average salary of pathology technician is 717 dollars   § Programmatic cost was 48% of direct medical cost. 23% for quality control and training and 25% for administration and recruitment  International dollars exchange using 2013 purchasing power parity (PPP) exchange rate (1 I$ = 2,694.27 kips) [[31](#_ENREF_31)] | | | |

**Table 9: Detail of laboratory cost**

| **Item** | **Sub-item** | **Unit price (dollar)** | **Source** |
| --- | --- | --- | --- |
| Conventional cervical cytology | Lab administration ‡ | 0.01 | Personal communication with a head of department of Pathology center, University of Health Science, Ministry of Health, Lao PDR |
|  | Lab equipment # | 11.20 |  |
|  | Lab stuffs ¶ | 3.54 |  |
|  | **Total** | 14.75 |  |
| Liquid-based cervical cytology | Lab administration ‡ | 0.01 |  |
|  | Lab equipment # | 20.96 |  |
|  | Lab stuffs ¶ | 4.55 |  |
|  | **Total** | 25.52 |  |
| Histology | Lab administration ‡ | 14.48 |  |
|  | Lab equipment * | 15.47 |  |
|  | Lab stuffs ¶ | 15.74 |  |
|  | **Total** | 45.69 |  |
| **Note:**  # Consumable items included Brush, cover glass, Malinol, Gill hemato, OG-6, EA-50, mask, xytene, etanol, slide. LBC prep set, LBC liquid were added for Thin-Prep.  * Consumable items included Formaline, hematocyline , eosine, paraphine, casette, cyline, obsolute, acetone, malinone  ‡ Lab administration was retrieved from general administration allocated to laboratory in hospital per sample.  ¶ This included both technical stuff and pathologist cost. Each cost is calculated by multiplying time spending to procedure with labor cost per hour  International dollars exchange using purchasing power parity (PPP) exchange rate (1 I$ = 2,694.27 kips) [[31](#_ENREF_31)] | | | |

**Table 10: Costing of precancerous treatment**

| **Item** | **Sub-item** | Unit price (dollar) | **Source** |
| --- | --- | --- | --- |
| Cryotherapy | Administration ‡ | 10.66 | Personal communication with a head of department of health insurance, Ministry of health, Lao PDR |
|  | Drug and equipment cost * | 5.41 |  |
|  | Labor cost ‡‡ | 7.52 |  |
|  | **Total** # | 23.59 |  |
| LEEP | Administration ‡ | 27.66 |  |
|  | Drug and equipment cost * | 57.05 |  |
|  | Labor cost ‡‡ | 35.68 |  |
|  | **Total** # | 120.40 |  |
| Hysterectomy | Administration † | 64.63 |  |
|  | Drug and medical equipment cost † | 204.23 |  |
|  | Labor cost † | 76.96 |  |
|  | **Subtotal** | 345.82 |  |
|  | Hospitalisation cost in 7 days § | 842.78 |  |
|  | **Total** # | 1188.59 |  |
| Cancer treatment §§ | Treatment cost of Local cancer | 745.57 (372.79-1491.15) | [[33](#_ENREF_33)] |
|  | Treatment cost of regional cancer | 845.68 (422.85-1691.36) |  |
|  | Treatment cost of distant cancer | 845.68 (422.85-1691.36) |  |
| **Note:**  ‡ Administration included general and medical administration. General administration included electricity, water and transportation supplies and other office martials and stuffs. Medical administration included training support and aids, some medical equipment; outpatient administration for cryotherapy and inpatient for loop electrosurgical excision procedure (LEEP).  ‡‡ Labor cost was calculated by multiplying the wage rate per hour by the time spent to provide treatment  * Drug and equipment cost consist of the average cost per patient of in and out clinics.  † Due to lack of data specific to obstetric surgery, administration, drug and medical equipment and labor cost of hysterectomy an average cost of a surgery case at the department of gyneco-obstetrics in Mahosot and Setthathirath hospitals was used.  § Hospitalization cost consists of the average cost of hospitalization per day at the department of gyneco-obstetric in Mahosot and Setthathirath hospitals. We assumed that a patient was hospitalized for seven days  ≠ Total cost did not include the cost of follow-up for precancerous lesion because, according to expert, patients are lost at follow-up.  §§ Cost is unit price per person, 2013 International dollars exchange using purchasing power parity (PPP) exchange rate (1 I$ = 2,694.27 kips) [[31](#_ENREF_31)] and the price of cancer treatment was adjusted from 2005 to 2014 using consumer price index (77.33 in 2005 and 122.52 in 2014) [[1](#_ENREF_1)] | | | |

**Result**

| **Table 11: Base case analyses of cost-effectiveness of prevention strategies against cervical cancer in women in Lao PDR (two-doses vaccination scenarios)** | | | | | | | | | | | | | |
| --- | --- | --- | --- | --- | --- | --- | --- | --- | --- | --- | --- | --- | --- |
| **Option** | **Cancer per 1,000 women** | **Cancer reduction per 1,000 women (N)** | **Cancer reduction (%)** | **DALY averted per 1,00 women** | **DALY averted per 1,000 women** | **Cost of screening and treatment per 1,000 women** | **Cost of cancer treatment per 1,000 women** | **Cost of vaccination per 1,000 women** | **Total cost per 1,000 women** | **CER (cancer)** | **CER (DALY averted)** | **ICER (cancer reduction)** | **ICER (DALY averted)** |
| Baseline | 4.8 | Ref | Ref | 57.9 | Ref | 3940 | 776 | 0 | 4716 | - | - | - | - |
| Five-yearly VIA_30-65 | 2.7 | 2.1 | 56.5 | 33.4 | 24.5 | 12823 | 502 | 0 | 13325 | 6448 | 544 | 4166 | 351 |
| Five-yearly VIA_25-65 | 2.5 | 2.3 | 52.3 | 30.9 | 27.0 | 15119 | 479 | 0 | 15598 | 6878 | 577 | 11301 | 895 |
| Five-yearly VIA_20-65 | 2.3 | 2.4 | 48.8 | 28.8 | 29.1 | 17470 | 461 | 0 | 17932 | 7370 | 616 | 14105 | 1135 |
| Vaccination | 2.2 | 2.6 | 45.5 | 27.5 | 30.5 | 3904 | 527 | 15561 | 19989 | 7716 | 656 | 13086 | 1500 |
| Three-yearly VIA_30-65 | 2.0 | 2.8 | 42.1 | 25.1 | 32.8 | 21362 | 404 | 0 | 21766 | 7913 | 663 | 11086 | 750 |
| Five-yearly cytology_30-65 | 3.7 | 1.1 | 76.9 | 44.1 | 13.8 | 22048 | 654 | 0 | 22701 | 20659 | 1647 | D | D |
| Five-yearly combined testing_30-65 | 3.7 | 1.1 | 76.9 | 44.1 | 13.8 | 23195 | 654 | 0 | 23848 | 21703 | 1730 | D | D |
| Five-yearly rapid HPV DNA_30-65 | 3.3 | 1.4 | 70.4 | 40.2 | 17.7 | 23268 | 611 | 0 | 23879 | 16964 | 1350 | D | D |
| Three-yearly VIA_25-65 | 1.8 | 2.9 | 38.0 | 22.6 | 35.3 | 25186 | 380 | 0 | 25566 | 8672 | 723 | D | D |
| Five-yearly cytology_25-65 | 3.5 | 1.3 | 73.4 | 42.1 | 15.8 | 25933 | 636 | 0 | 26568 | 21037 | 1678 | D | D |
| Five-yearly combined testing_25-65 | 3.5 | 1.3 | 73.4 | 42.1 | 15.8 | 27285 | 636 | 0 | 27921 | 22108 | 1764 | D | D |
| Five-yearly rapid HPV DNA_25-65 | 3.2 | 1.6 | 66.6 | 38.0 | 19.9 | 27356 | 591 | 0 | 27947 | 17585 | 1401 | D | D |
| Five-yearly VIA_30-65 + vaccination | 1.4 | 3.3 | 30.3 | 18.6 | 39.3 | 12817 | 368 | 15555 | 28740 | 8679 | 731 | 12441 | 1078 |
| Three-yearly VIA_20-65 | 1.7 | 3.1 | 34.8 | 20.7 | 37.2 | 29102 | 362 | 0 | 29464 | 9500 | 791 | D | D |
| Five-yearly liquid_30-65 | 3.7 | 1.1 | 76.9 | 44.1 | 13.8 | 28950 | 654 | 0 | 29604 | 26941 | 2148 | D | D |
| Five-yearly cytology_20-65 | 3.3 | 1.4 | 70.5 | 40.4 | 17.6 | 29922 | 621 | 0 | 30542 | 21748 | 1739 | D | D |
| Five-yearly VIA_25-65 + vaccination | 1.4 | 3.3 | 29.5 | 18.1 | 39.8 | 15112 | 360 | 15554 | 31025 | 9264 | 779 | ED | ED |
| Five-yearly combined testing_20-65 | 3.3 | 1.4 | 70.5 | 40.4 | 17.6 | 31485 | 621 | 0 | 32105 | 22861 | 1828 | D | D |
| Five-yearly rapid HPV DNA_20-65 | 3.0 | 1.7 | 63.3 | 36.1 | 21.8 | 31554 | 574 | 0 | 32128 | 18424 | 1472 | D | D |
| Five-yearly VIA_20-65 + vaccination | 1.4 | 3.4 | 29.0 | 17.7 | 40.2 | 17461 | 354 | 15551 | 33366 | 9881 | 829 | ED | ED |
| Five-yearly liquid_25-65 | 3.5 | 1.3 | 73.4 | 42.1 | 15.8 | 34073 | 636 | 0 | 34708 | 27483 | 2192 | D | D |
| Three-yearly cytology_30-65 | 3.0 | 1.7 | 63.4 | 36.2 | 21.8 | 36622 | 577 | 0 | 37199 | 21390 | 1709 | D | D |
| Three-yearly VIA_30-65 + vaccination | 1.2 | 3.6 | 24.8 | 15.3 | 42.6 | 21354 | 309 | 15551 | 37214 | 10405 | 873 | 31945 | 2544 |
| Five-yearly cytology_30-65 + vaccination | 1.8 | 3.0 | 37.5 | 22.4 | 35.6 | 21917 | 458 | 15560 | 37934 | 12769 | 1067 | D | D |
| Five-yearly combined testing_30-65 + vaccination | 1.8 | 3.0 | 37.5 | 22.4 | 35.6 | 23063 | 458 | 15560 | 39081 | 13155 | 1099 | D | D |
| Three-yearly combined testing_30-65 | 3.0 | 1.7 | 63.4 | 36.2 | 21.8 | 38533 | 577 | 0 | 39110 | 22489 | 1797 | D | D |
| Five-yearly rapid HPV DNA_30-65 + vaccination | 1.7 | 3.1 | 35.2 | 20.9 | 37.1 | 23120 | 432 | 15560 | 39113 | 12698 | 1055 | D | D |
| Three-yearly rapid HPV DNA_30-65 | 2.7 | 2.1 | 56.2 | 31.8 | 26.1 | 38612 | 528 | 0 | 39140 | 18792 | 1500 | D | D |
| Five-yearly liquid_20-65 | 3.3 | 1.4 | 70.5 | 40.4 | 17.6 | 39329 | 621 | 0 | 39950 | 28447 | 2274 | D | D |
| Three-yearly VIA_25-65 + vaccination | 1.1 | 3.6 | 23.9 | 14.7 | 43.2 | 25176 | 300 | 15549 | 41025 | 11344 | 949 | 486 | 6735 |
| Five-yearly cytology_25-65 + vaccination | 1.8 | 3.0 | 36.9 | 22.0 | 36.0 | 25798 | 451 | 15560 | 41810 | 13936 | 1162 | D | D |
| Five-yearly combined testing_25-65 + vaccination | 1.8 | 3.0 | 36.9 | 22.0 | 36.0 | 27151 | 451 | 15560 | 43162 | 14386 | 1200 | D | D |
| Five-yearly rapid HPV DNA_25-65 + vaccination | 1.6 | 3.1 | 34.5 | 20.4 | 37.5 | 27208 | 425 | 15560 | 43193 | 13876 | 1151 | D | D |
| Three-yearly cytology_25-65 | 2.8 | 1.9 | 59.2 | 33.7 | 24.2 | 43082 | 554 | 0 | 43636 | 22517 | 1801 | D | D |
| Five-yearly liquid_30-65 + vaccination | 1.8 | 3.0 | 37.5 | 22.4 | 35.6 | 28818 | 458 | 15560 | 44836 | 15092 | 1261 | D | D |
| Three-yearly VIA_20-65 + vaccination | 1.1 | 3.6 | 23.3 | 14.3 | 43.6 | 29090 | 293 | 15544 | 44927 | 12321 | 1030 | 130720 | 9427 |
| Five-yearly cytology_20-65 + vaccination | 1.7 | 3.0 | 36.4 | 21.7 | 36.3 | 29783 | 447 | 15560 | 45790 | 15152 | 1262 | D | D |
| Three-yearly combined testing_25-65 | 2.8 | 1.9 | 59.2 | 33.7 | 24.2 | 45336 | 554 | 0 | 45890 | 23680 | 1894 | D | D |
| Three-yearly rapid HPV DNA_25-65 | 2.5 | 2.3 | 51.8 | 29.3 | 28.6 | 45408 | 504 | 0 | 45912 | 20057 | 1604 | D | D |
| Five-yearly combined testing_20-65 + vaccination | 1.7 | 3.0 | 36.4 | 21.7 | 36.3 | 31346 | 447 | 15560 | 47353 | 15669 | 1306 | D | D |
| Five-yearly rapid HPV DNA_20-65 + vaccination | 1.6 | 3.1 | 34.0 | 20.1 | 37.8 | 31404 | 420 | 15560 | 47384 | 15105 | 1252 | D | D |
| Three-yearly liquid_30-65 | 3.0 | 1.7 | 63.4 | 36.2 | 21.8 | 48124 | 577 | 0 | 48701 | 28005 | 2237 | D | D |
| Five-yearly liquid_25-65 + vaccination | 1.8 | 3.0 | 36.9 | 22.0 | 36.0 | 33937 | 451 | 15560 | 49949 | 16649 | 1388 | D | D |
| Three-yearly cytology_20-65 | 2.6 | 2.1 | 55.7 | 31.7 | 26.2 | 49717 | 536 | 0 | 50253 | 23888 | 1916 | D | D |
| Three-yearly cytology_30-65 + vaccination | 1.6 | 3.2 | 32.9 | 19.5 | 38.5 | 36451 | 416 | 15559 | 52426 | 16443 | 1363 | D | D |
| Three-yearly combined testing_20-65 | 2.6 | 2.1 | 55.7 | 31.7 | 26.2 | 52322 | 536 | 0 | 52857 | 25126 | 2015 | D | D |
| Three-yearly rapid HPV DNA_20-65 | 2.3 | 2.5 | 48.3 | 27.3 | 30.6 | 52390 | 484 | 0 | 52875 | 21514 | 1726 | D | D |
| Three-yearly combined testing_30-65 + vaccination | 1.6 | 3.2 | 32.9 | 19.5 | 38.5 | 38362 | 416 | 15559 | 54337 | 17043 | 1413 | D | D |
| Three-yearly rapid HPV DNA_30-65 + vaccination | 1.4 | 3.3 | 30.2 | 17.7 | 40.2 | 38429 | 387 | 15559 | 54375 | 16396 | 1353 | D | D |
| Five-yearly liquid_20-65 + vaccination | 1.7 | 3.0 | 36.4 | 21.7 | 36.3 | 39190 | 447 | 15560 | 55196 | 18265 | 1522 | D | D |
| Three-yearly liquid_25-65 | 2.8 | 1.9 | 59.2 | 33.7 | 24.2 | 56647 | 554 | 0 | 57201 | 29517 | 2361 | D | D |
| Three-yearly cytology_25-65 + vaccination | 1.5 | 3.2 | 32.2 | 19.0 | 39.0 | 42914 | 408 | 15559 | 58881 | 18258 | 1511 | D | D |
| Three-yearly combined testing_25-65 + vaccination | 1.5 | 3.2 | 32.2 | 19.0 | 39.0 | 45167 | 408 | 15559 | 61134 | 18957 | 1569 | D | D |
| Three-yearly rapid HPV DNA_25-65 + vaccination | 1.4 | 3.4 | 29.4 | 17.2 | 40.7 | 45233 | 378 | 15559 | 61170 | 18234 | 1502 | D | D |
| Three-yearly liquid_30-65 + vaccination | 1.6 | 3.2 | 32.9 | 19.5 | 38.5 | 47953 | 416 | 15559 | 63929 | 20051 | 1662 | D | D |
| Three-yearly cytology_20-65 + vaccination | 1.5 | 3.3 | 31.6 | 18.6 | 39.3 | 49550 | 402 | 15559 | 65511 | 20146 | 1666 | D | D |
| Three-yearly liquid_20-65 | 2.6 | 2.1 | 55.7 | 31.7 | 26.2 | 65395 | 536 | 0 | 65930 | 31340 | 2513 | D | D |
| Three-yearly combined testing_20-65 + vaccination | 1.5 | 3.3 | 31.6 | 18.6 | 39.3 | 52154 | 402 | 15559 | 68115 | 20947 | 1732 | D | D |
| Three-yearly rapid HPV DNA_20-65 + vaccination | 1.4 | 3.4 | 28.8 | 16.8 | 41.1 | 52221 | 372 | 15559 | 68151 | 20145 | 1658 | D | D |
| Three-yearly liquid_25-65 + vaccination | 1.5 | 3.2 | 32.2 | 19.0 | 39.0 | 56478 | 408 | 15559 | 72445 | 22465 | 1859 | D | D |
| Three-yearly liquid_20-65 + vaccination | 1.5 | 3.3 | 31.6 | 18.6 | 39.3 | 65226 | 402 | 15559 | 81187 | 24967 | 2064 | D | D |
| **Note:**  The analyses did not include the yearly screening options.  Baseline refers to no vaccination with 5.2% cytology screening for women aged 18-68 years old.  Vaccination is two-doses for 10-years-old girls. Cytology refers to conventional cervical cytology; LBC refers to liquid-based cervical cytology; HPV testing refers to rapid HPV DNA testing; VIA+cytology refers to the combined testing VIA and cytology.  The incremental cost of effectiveness ratio expressed as cancer prevented or DALY averted is listed in order of increasing cost. In non-dominant strategy, the ICER was calculated by devising different cost to different effectiveness.  **D** refers to strong dominance, which is expressed as higher cost, but lower effectiveness than alternative options.  **ED** refers to extendedly dominance, which has higher ICER than the next ICER. | | | | | | | | | | | | | |

| **Table 12: Base case analyses of cost-effectiveness of prevention strategies against cervical cancer in women in Lao PDR (three-doses vaccination scenarios)** | | | | | | | | | | | | | |
| --- | --- | --- | --- | --- | --- | --- | --- | --- | --- | --- | --- | --- | --- |
| **Option** | **Cancer per 1,000 women** | **Cancer reduction per 1,000 women (N)** | **Cancer reduction (%)** | **DALY averted per 1,00 women** | **DALY averted per 1,000 women** | **Cost of screening and treatment per 1,000 women** | **Cost of cancer treatment per 1,000 women** | **Cost of vaccination per 1,000 women** | **Total cost per 1,000 women** | **CER (cancer)** | **CER (DALY averted)** | **ICER (cancer reduction)** | **ICER (DALY averted)** |
| Baseline | 4.8 | Ref | Ref | 57.9 | Ref | 3940 | 776 | 0 | 4716 | - | - | - | - |
| VIA alone_30-65 | 2.7 | 2.1 | 43.5 | 33.4 | 24.5 | 12823 | 502 | 0 | 13325 | 6448 | 544 | 4166 | 351 |
| Five-yearly VIA alone_25-65 | 2.5 | 2.3 | 47.7 | 30.9 | 27 | 15119 | 479 | 0 | 15598 | 6878 | 577 | 11302 | 895 |
| Five-yearly VIA alone_20-65 | 2.3 | 2.4 | 51.2 | 28.8 | 29.1 | 17470 | 461 | 0 | 17932 | 7370 | 616 | ED | ED |
| Three-yearly VIA alone_30-65 | 2 | 2.8 | 57.9 | 25.1 | 32.8 | 21362 | 404 | 0 | 21766 | 7913 | 663 | 12771 | 1064 |
| Vaccination | 2.1 | 2.6 | 54.9 | 27.2 | 30.7 | 3901 | 524 | 17399 | 21824 | 8362 | 710 | D | D |
| Five-yearly conventional cytology_30-65 | 3.7 | 1.1 | 23.1 | 44.1 | 13.8 | 22048 | 654 | 0 | 22701 | 20659 | 1647 | D | D |
| Five-yearly rapid HPV DNA testing_30-65 | 2.9 | 1.9 | 39.1 | 34.6 | 23.4 | 22217 | 547 | 0 | 22764 | 12254 | 974 | D | D |
| Five-yearly combined VIA and cytology testing_30-65 | 3.1 | 1.6 | 33.8 | 37.7 | 20.2 | 24938 | 583 | 0 | 25521 | 15865 | 1262 | D | D |
| Three-yearly VIA alone_25-65 | 1.8 | 2.9 | 62 | 22.6 | 35.3 | 25186 | 380 | 0 | 25566 | 8672 | 723 | ED | ED |
| Five-yearly conventional cytology_25-65 | 3.5 | 1.3 | 26.6 | 42.1 | 15.8 | 25933 | 636 | 0 | 26568 | 21037 | 1678 | D | D |
| Five-yearly rapid HPV DNA testing_25-65 | 2.7 | 2.1 | 43.3 | 32.1 | 25.8 | 26093 | 524 | 0 | 26618 | 12946 | 1031 | D | D |
| Three-yearly VIA alone_20-65 | 1.7 | 3.1 | 65.2 | 20.7 | 37.2 | 29102 | 362 | 0 | 29464 | 9500 | 791 | ED | ED |
| Five-yearly combined VIA and cytology testing_25-65 | 3 | 1.8 | 37.8 | 35.3 | 22.6 | 29315 | 562 | 0 | 29877 | 16607 | 1323 | D | D |
| Five-yearly liquid-based cytology_30-65 | 3.1 | 1.6 | 34.2 | 37.5 | 20.4 | 29748 | 581 | 0 | 30329 | 18669 | 1485 | D | D |
| Five-yearly conventional cytology_20-65 | 3.3 | 1.4 | 29.5 | 40.4 | 17.6 | 29922 | 621 | 0 | 30542 | 21748 | 1739 | D | D |
| VIA alone_30-65 + vaccination | 1.4 | 3.3 | 69.7 | 18.6 | 39.3 | 12817 | 368 | 17392 | 30577 | 9234 | 778 | 15718 | 1362 |
| Five-yearly rapid HPV DNA testing_20-65 | 2.5 | 2.2 | 46.7 | 30.1 | 27.8 | 30081 | 506 | 0 | 30587 | 13770 | 1100 | D | D |
| Five-yearly VIA alone_25-65 + vaccination | 1.4 | 3.3 | 70.5 | 18.1 | 39.8 | 15112 | 360 | 17391 | 32862 | 9813 | 825 | D | ED |
| Five-yearly combined VIA and cytology testing_20-65 | 2.8 | 2 | 41.2 | 33.4 | 24.5 | 33813 | 544 | 0 | 34357 | 17532 | 1400 | D | D |
| Five-yearly VIA alone_20-65 + vaccination | 1.4 | 3.4 | 71 | 17.7 | 40.2 | 17461 | 354 | 17387 | 35202 | 10424 | 875 | ED | ED |
| Five-yearly liquid-based cytology_25-65 | 2.9 | 1.8 | 38.2 | 35.1 | 22.8 | 34987 | 559 | 0 | 35546 | 19578 | 1559 | D | D |
| Three-yearly conventional cytology_30-65 | 3 | 1.7 | 36.6 | 36.2 | 21.8 | 36622 | 577 | 0 | 37199 | 21390 | 1709 | D | D |
| Three-yearly rapid HPV DNA testing_30-65 | 2.2 | 2.5 | 53.5 | 26.1 | 31.8 | 36783 | 459 | 0 | 37242 | 14654 | 1170 | D | D |
| Three-yearly VIA alone_30-65 + vaccination | 1.2 | 3.6 | 75.2 | 15.3 | 42.6 | 21354 | 309 | 17388 | 39051 | 10919 | 916 | 4468 | 2544 |
| Five-yearly conventional cytology_30-65 + vaccination | 1.8 | 3 | 62.5 | 22.4 | 35.6 | 21917 | 458 | 17398 | 39772 | 13387 | 1118 | D | D |
| Five-yearly rapid HPV DNA testing_30-65 + vaccination | 1.5 | 3.2 | 68.2 | 18.6 | 39.3 | 22047 | 393 | 17398 | 39837 | 12280 | 1014 | D | D |
| Five-yearly liquid-based cytology_20-65 | 2.8 | 2 | 41.6 | 33.2 | 24.7 | 40368 | 542 | 0 | 40910 | 20697 | 1653 | D | D |
| Three-yearly combined VIA and cytology testing_30-65 | 2.5 | 2.3 | 48.3 | 29.2 | 28.7 | 41361 | 497 | 0 | 41858 | 18249 | 1457 | D | D |
| Five-yearly combined VIA and cytology testing_30-65 + vaccination | 1.6 | 3.2 | 66.3 | 19.9 | 38 | 24779 | 415 | 17398 | 42592 | 13509 | 1119 | D | D |
| Three-yearly VIA alone_25-65 + vaccination | 1.1 | 3.6 | 76.1 | 14.7 | 43.2 | 25176 | 300 | 17386 | 42862 | 11852 | 992 | D | ED |
| Three-yearly conventional cytology_25-65 | 2.8 | 1.9 | 40.8 | 33.7 | 24.2 | 43082 | 554 | 0 | 43636 | 22517 | 1801 | D | D |
| Five-yearly conventional cytology_25-65 + vaccination | 1.8 | 3 | 63.1 | 22 | 36 | 25798 | 451 | 17398 | 43647 | 14548 | 1213 | D | D |
| Three-yearly rapid HPV DNA testing_25-65 | 2 | 2.7 | 57.8 | 23.6 | 34.3 | 43225 | 434 | 0 | 43659 | 15900 | 1272 | D | D |
| Five-yearly rapid HPV DNA testing_25-65 + vaccination | 1.5 | 3.3 | 69 | 18.1 | 39.8 | 25927 | 385 | 17398 | 43710 | 13326 | 1098 | D | D |
| Three-yearly VIA alone_20-65 + vaccination | 1.1 | 3.6 | 76.7 | 14.3 | 43.6 | 29090 | 293 | 17380 | 46763 | 12825 | 1072 | D | ED |
| Five-yearly combined VIA and cytology testing_25-65 | 1.6 | 3.2 | 67 | 19.4 | 38.5 | 29158 | 407 | 17398 | 46963 | 14736 | 1219 | D | D |
| Five-yearly liquid-based cytology_30-65 + vaccination | 1.6 | 3.2 | 66.4 | 19.8 | 38.1 | 29587 | 414 | 17398 | 47399 | 15007 | 1243 | D | D |
| Five-yearly conventional cytology_20-65 + vaccination | 1.7 | 3 | 63.6 | 21.7 | 36.3 | 29783 | 447 | 17398 | 47628 | 15760 | 1313 | D | D |
| Five-yearly rapid HPV DNA testing_20-65 + vaccination | 1.4 | 3.3 | 69.6 | 17.8 | 40.2 | 29918 | 379 | 17397 | 47694 | 14424 | 1188 | D | D |
| Three-yearly combined VIA and cytology testing_25-65 | 2.3 | 2.5 | 52.6 | 26.7 | 31.3 | 48639 | 472 | 0 | 49111 | 19637 | 1571 | D | D |
| Three-yearly liquid-based cytology_30-65 | 2.4 | 2.3 | 48.6 | 29 | 28.9 | 49374 | 495 | 0 | 49868 | 21588 | 1723 | D | D |
| Three-yearly conventional cytology_20-65 | 2.6 | 2.1 | 44.3 | 31.7 | 26.2 | 49717 | 536 | 0 | 50253 | 23888 | 1916 | D | D |
| Three-yearly rapid HPV DNA testing_20-65 | 1.8 | 2.9 | 61.2 | 21.7 | 36.2 | 49856 | 414 | 0 | 50270 | 17291 | 1388 | D | D |
| Five-yearly combined VIA and cytology testing_20-65 + vaccination | 1.5 | 3.2 | 67.6 | 19.1 | 38.9 | 33655 | 402 | 17397 | 51455 | 16018 | 1324 | D | D |
| Five-yearly liquid-based cytology_25-65 + vaccination | 1.6 | 3.2 | 67.2 | 19.3 | 38.6 | 34827 | 406 | 17398 | 52631 | 16484 | 1363 | D | D |
| Three-yearly conventional cytology_30-65 + vaccination | 1.6 | 3.2 | 67.1 | 19.5 | 38.5 | 36451 | 416 | 17397 | 54264 | 17020 | 1411 | D | D |
| Three-yearly rapid HPV DNA testing_30-65 + vaccination | 1.3 | 3.5 | 73.5 | 15.3 | 42.6 | 36586 | 344 | 17397 | 54327 | 15542 | 1275 | D | D |
| Three-yearly combined VIA and cytology testing_20-65 | 2.1 | 2.7 | 56.1 | 24.7 | 33.3 | 56119 | 452 | 0 | 56571 | 21205 | 1701 | D | D |
| Five-yearly liquid-based cytology_20-65 + vaccination | 1.5 | 3.2 | 67.7 | 19 | 39 | 40208 | 401 | 17397 | 58007 | 18024 | 1489 | D | D |
| Three-yearly liquid-based cytology_25-65 | 2.2 | 2.5 | 53 | 26.5 | 31.5 | 58086 | 470 | 0 | 58556 | 23263 | 1861 | D | D |
| Three-yearly combined VIA and cytology testing_30-65 + vaccination | 1.4 | 3.4 | 71.5 | 16.7 | 41.3 | 41170 | 368 | 17397 | 58935 | 17348 | 1428 | D | D |
| Three-yearly conventional cytology_25-65 + vaccination | 1.5 | 3.2 | 67.8 | 19 | 39 | 42914 | 408 | 17397 | 60718 | 18828 | 1558 | D | D |
| Three-yearly rapid HPV DNA testing_25-65 + vaccination | 1.2 | 3.5 | 74.4 | 14.8 | 43.1 | 43044 | 334 | 17397 | 60775 | 17193 | 1409 | D | D |
| Yearly VIA alone_30-65 | 1 | 3.8 | 79.9 | 12.2 | 45.7 | 64028 | 234 | 0 | 64261 | 16929 | 1405 | ED | ED |
| Three-yearly combined VIA and cytology testing_25-65 + vaccination | 1.3 | 3.4 | 72.3 | 16.1 | 41.8 | 48459 | 358 | 17397 | 66214 | 19268 | 1584 | D | D |
| Three-yearly liquid-based cytology_30-65 + vaccination | 1.3 | 3.4 | 71.6 | 16.6 | 41.4 | 49181 | 366 | 17397 | 66944 | 19668 | 1619 | D | D |
| Three-yearly conventional cytology_20-65 + vaccination | 1.5 | 3.3 | 68.4 | 18.6 | 39.3 | 49550 | 402 | 17397 | 67348 | 20711 | 1713 | D | D |
| Three-yearly rapid HPV DNA testing_20-65 + vaccination | 1.2 | 3.6 | 75 | 14.4 | 43.5 | 49687 | 328 | 17396 | 67411 | 18914 | 1549 | D | D |
| Three-yearly liquid-based cytology_20-65 | 2.1 | 2.7 | 56.5 | 24.5 | 33.5 | 67039 | 450 | 0 | 67488 | 25147 | 2018 | D | D |
| Three-yearly combined VIA and cytology testing_20-65 + vaccination | 1.3 | 3.5 | 72.9 | 15.7 | 42.2 | 55947 | 352 | 17396 | 73696 | 21266 | 1746 | D | D |
| Three-yearly liquid-based cytology_25-65 + vaccination | 1.3 | 3.4 | 72.4 | 16 | 41.9 | 57905 | 357 | 17397 | 75658 | 21976 | 1806 | D | D |
| Yearly VIA alone_25-65 | 0.8 | 3.9 | 82.3 | 10.7 | 47.2 | 75484 | 216 | 0 | 75700 | 19343 | 1603 | ED | ED |
| Yearly VIA alone_30-65 + vaccination | 0.7 | 4.1 | 85.7 | 9 | 49 | 64008 | 195 | 17372 | 81575 | 20013 | 1666 | 85116 | 6733 |
| Three-yearly liquid-based cytology_20-65 + vaccination | 1.3 | 3.5 | 73 | 15.6 | 42.3 | 66866 | 351 | 17396 | 84613 | 24372 | 2001 | D | D |
| Yearly VIA alone_20-65 | 0.8 | 4 | 84 | 9.7 | 48.2 | 87213 | 204 | 0 | 87417 | 21885 | 1813 | D | D |
| Yearly VIA alone_25-65 + vaccination | 0.6 | 4.1 | 86.5 | 8.5 | 49.4 | 75450 | 187 | 17365 | 93002 | 22631 | 1881 | D | 24136 |
| Yearly VIA alone_20-65 + vaccination | 0.6 | 4.1 | 87 | 8.1 | 49.8 | 87151 | 181 | 17350 | 104683 | 25303 | 2101 | 422480 | 30462 |
| Yearly rapid HPV DNA testing_30-65 | 1.1 | 3.7 | 77.5 | 12.3 | 45.7 | 108925 | 283 | 0 | 109208 | 29660 | 2391 | D | D |
| Yearly conventional cytology_30-65 | 1.6 | 3.2 | 66.5 | 18.6 | 39.3 | 108931 | 381 | 0 | 109312 | 34604 | 2782 | D | D |
| Yearly combined VIA and cytology testing_30-65 | 1.2 | 3.5 | 74.5 | 14 | 44 | 122813 | 311 | 0 | 123124 | 34771 | 2801 | D | D |
| Yearly rapid HPV DNA testing_30-65 + vaccination | 0.7 | 4 | 84.4 | 8.8 | 49.2 | 108742 | 234 | 17395 | 126370 | 31501 | 2571 | D | D |
| Yearly conventional cytology_30-65 + vaccination | 1 | 3.7 | 78.8 | 12.2 | 45.8 | 108727 | 302 | 17395 | 126424 | 33731 | 2763 | D | D |
| Yearly rapid HPV DNA testing_25-65 | 0.9 | 3.8 | 80.2 | 10.7 | 47.2 | 128229 | 263 | 0 | 128492 | 33711 | 2723 | D | D |
| Yearly conventional cytology_25-65 | 1.4 | 3.3 | 70.2 | 16.5 | 41.4 | 128256 | 356 | 0 | 128612 | 38529 | 3105 | D | D |
| Yearly combined VIA and cytology testing_30-65 + vaccination | 0.8 | 3.9 | 82.8 | 9.7 | 48.2 | 122624 | 254 | 17395 | 140273 | 35639 | 2911 | D | D |
| Yearly combined VIA and cytology testing_25-65 | 1.1 | 3.7 | 77.5 | 12.3 | 45.7 | 144608 | 290 | 0 | 144898 | 39310 | 3173 | D | D |
| Yearly rapid HPV DNA testing_25-65 + vaccination | 0.7 | 4 | 85.1 | 8.3 | 49.6 | 128081 | 225 | 17395 | 145701 | 36014 | 2937 | D | D |
| Yearly conventional cytology_25-65 + vaccination | 1 | 3.8 | 79.7 | 11.6 | 46.3 | 128080 | 292 | 17395 | 145767 | 38487 | 3149 | D | D |
| Yearly liquid-based cytology_30-65 | 1.2 | 3.6 | 74.7 | 13.8 | 44.1 | 146828 | 309 | 0 | 147137 | 41439 | 3338 | D | D |
| Yearly rapid HPV DNA testing_20-65 | 0.9 | 3.9 | 82.1 | 9.7 | 48.2 | 148098 | 248 | 0 | 148346 | 38024 | 3077 | D | D |
| Yearly conventional cytology_20-65 | 1.3 | 3.5 | 73 | 15 | 42.9 | 148114 | 338 | 0 | 148452 | 42787 | 3457 | D | D |
| Yearly combined VIA and cytology testing_25-65 + vaccination | 0.8 | 4 | 83.6 | 9.3 | 48.7 | 144453 | 245 | 17395 | 162093 | 40811 | 3331 | D | D |
| Yearly liquid-based cytology_30-65 + vaccination | 0.8 | 3.9 | 82.9 | 9.7 | 48.2 | 146639 | 253 | 17395 | 164287 | 41686 | 3405 | D | D |
| Yearly rapid HPV DNA testing_20-65 + vaccination | 0.7 | 4.1 | 85.7 | 16.7 | 41.3 | 147975 | 219 | 17394 | 165588 | 40650 | 3314 | D | D |
| Yearly conventional cytology_20-65 + vaccination | 0.9 | 3.8 | 80.3 | 11.3 | 46.7 | 147959 | 285 | 17395 | 165638 | 43392 | 3548 | D | D |
| Yearly combined VIA and cytology testing_20-65 | 1 | 3.8 | 79.7 | 11.1 | 46.8 | 167016 | 274 | 0 | 167290 | 44165 | 3572 | D | D |
| Yearly liquid-based cytology_25-65 | 1.1 | 3.7 | 77.7 | 12.2 | 45.8 | 172928 | 288 | 0 | 173216 | 46882 | 3784 | D | D |
| Yearly combined VIA and cytology testing_20-65 + vaccination | 0.8 | 4 | 84.2 | 8.9 | 49 | 166884 | 238 | 17394 | 184517 | 46126 | 3763 | D | D |
| Yearly liquid-based cytology_25-65 + vaccination | 0.8 | 4 | 83.7 | 9.2 | 48.7 | 172773 | 244 | 17395 | 190411 | 47881 | 3907 | D | D |
| Yearly liquid-based cytology_20-65 | 1 | 3.8 | 79.8 | 11 | 46.9 | 199751 | 272 | 0 | 200023 | 52697 | 4263 | D | D |
| Yearly liquid-based cytology_20-65 + vaccination | 0.7 | 4 | 84.3 | 8.8 | 49.1 | 199620 | 237 | 17394 | 217251 | 54242 | 4425 | D | D |
| **Note:**  The analyses included yearly, three-yearly and five-yearly screening options.  Baseline refers to no vaccination with 5.2% cytology screening for women aged 18-68 years old.  Vaccination is three-doses for 10-years-old girls. Cytology refers to conventional cervical cytology; LBC refers to liquid-based cervical cytology; HPV testing refers to rapid HPV DNA testing; VIA+cytology refers to the combined testing VIA and cytology.  The incremental cost of effectiveness ratio expressed as cancer prevented or DALY averted is listed in order of increasing cost. In non-dominant strategy, the ICER was calculated by devising different cost to different effectiveness.  **D** refers to strong dominance, which is expressed as higher cost, but lower effectiveness than alternative options.  **ED** refers to extendedly dominance, which has higher ICER than the next ICER. | | | | | | | | | | | | | |

**Sensitivity analysis**

| **Table 13: Univariate sensitivity analyses of impact of cost of vaccine and screening coverage on ICER per DALY averted by screening strategies** | | | | | | | | | | | | |
| --- | --- | --- | --- | --- | --- | --- | --- | --- | --- | --- | --- | --- |
| Options | Vaccination coverage (%)† | | | | | | Screening coverage (%)¶ | | | | | |
|  | 10 | 30 | 50 | 70 | 80 |  | 10 | 30 | 50 | 70 | 80 |  |
| Triennial VIA_30-65 Vs vaccination | 752 | 1670 | 2186 | D | D |  | 837 | 1648 | D | 952 | 1251 |  |
| Triennial VIA_30-65 + vaccination | 146 | 365 | 784 | 1763 | 2987 |  | 108 | 1019 | 1445 | 1826 | 2011 |  |
| Quinquennial VIA_30-65 + vaccination | 101 | 440 | 534 | 778 | 1088 |  | D | 658 | 1019 | 1284 | 1406 |  |
| Quinquennial conventional cytology_30-65 + vaccination | 68 | 160 | 345 | 784 | 1333 |  | D | 3504 | 3709 | 3995 | 4147 |  |
| Quinquennial liquid-based cytology_30-65 + vaccination | 79 | 194 | 423 | 965 | 1642 |  | 1736 | 2930 | 3455 | 3932 | 4166 |  |
| Quinquennial combined VIA and cytology_30-65 + vaccination | 78 | 193 | 420 | 958 | 1631 |  | 943 | 2351 | 2836 | 3252 | 3452 |  |
| Triennial rapid HPV DNA_30-65 + vaccination | 119 | 315 | 695 | 1588 | 2704 |  | 1060 | 2102 | 2738 | 3334 | 3628 |  |
| Quinquennial rapid HPV DNA_30-65 + vaccination | 86 | 215 | 470 | 1072 | 1826 |  | 285 | 1624 | 2102 | 2493 | 2678 |  |
| **Note:**  † For different vaccination coverage, the comparison is between combined screening with girl vaccination and screening alone  ¶ For different screening coverage, the comparison is between combined screening with girl vaccination and girl vaccination alone  D refers domination | | | | | | | | | | | |  |

| **Table 14: Univariate sensitivity analyses of impact of cost of vaccine and screening coverage on ICER per DALY averted by screening strategies** | | | | | | | | | | | | |
| --- | --- | --- | --- | --- | --- | --- | --- | --- | --- | --- | --- | --- |
| Options | Cost of vaccine per dose (I$)† | | | | | |  | | | | | |
|  | 4.5 | 10 | 30 | 50 | 70 | 100 |  |  |  |  |  |  |
| Triennial VIA_30-65 + vaccination | 1763 | 2451 | 3700 | 7447 | 9946 | 13694 |  |  |  |  |  |  |
| Quinquennial VIA_30-65 + vaccination | 1165 | 1620 | 2447 | 4929 | 6583 | 9065 |  |  |  |  |  |  |
| Quinquennial conventional cytology_30-65 + vaccination | 784 | 1093 | 1656 | 3343 | 4468 | 6156 |  |  |  |  |  |  |
| Quinquennial liquid-based cytology_30-65 + vaccination | 965 | 1345 | 2038 | 4114 | 5499 | 7576 |  |  |  |  |  |  |
| Quinquennial combined VIA and cytology_30-65 + vaccination | 958 | 1336 | 2024 | 4086 | 5461 | 7524 |  |  |  |  |  |  |
| Triennial rapid HPV DNA_30-65 + vaccination | 1588 | 2214 | 3352 | 6767 | 9044 | 12459 |  |  |  |  |  |  |
| Quinquennial rapid HPV DNA_30-65 + vaccination | 1072 | 1495 | 2265 | 4573 | 6112 | 8420 |  |  |  |  |  |  |
| **Note:**  † For different cost of vaccine, the comparison is between combined screening with girl vaccination and screening alone  D refers domination | | | | | | | | | | | |  |

| **Table 15: Univariate sensitivity analyses of impact of number of loss to follow-up and sensitivity of VIA on ICER per DALY averted by screening strategies** | | | | | | | |
| --- | --- | --- | --- | --- | --- | --- | --- |
| Options | Loss to follow-up (%) | | | Sensitivity of VIA (%)† | | | |
|  | 0 | 5 | 10 | 65.8 | 58.5 | 51.2 | 36.6 |
| Triennial VIA_30-65 Vs vaccination |  |  |  | D | 1221 | 477 | 182 |
| Quinquennial conventional cytology_30-65 + vaccination | 41757 | D | D | D | D | D | 23156 |
| Quinquennial liquid-based cytology_30-65 + vaccination | 5281 | 8515 | 31226 | D | 76554 | 14449 | 4469 |
| Quinquennial combined VIA and cytology_30-65 + vaccination | 3959 | 6449 | 26601 | D | 87116 | 10378 | 2813 |
| Triennial rapid HPV DNA_30-65 + vaccination | 5904 | 8386 | 16085 | 21156 | 8565 | 4669 | 1768 |
| Quinquennial rapid HPV DNA_30-65 + vaccination | 3502 | 5016 | 9619 | 12997 | 5484 | 3069 | 1222 |
| Triennial VIA_30-65 + vaccination¶ |  |  |  | 1667 | 1935 | 2269 | 3323 |
| Quinquennial VIA_30-65 + vaccination¶ |  |  |  | 1217 | 1462 | 1779 | 2850 |
| **Note:**  Except noted, all screening strategies are compared to VIA. Screening with 5-year interval is compared 5-year interval of VIA, and 3-year interval compared to 3-year interval of VIA.  All screening strategies are combined with girl vaccination, including VIA  † The sensitivity is assumed to be less than in base case in %.  ¶ The strategy is compared to vaccination alone  D refers domination | | | | | | | |

| **Table 16: Comparing the incremental cost/effectiveness ratio of liquid-based cytology by different sensitivity of conventional cytology** | | | | | |
| --- | --- | --- | --- | --- | --- |
| Sensitivity of conventional cytology | Baseline DALY per 1000 women | DALY per 1000 women | DALY averted | Cost per 1000 women | ICER (DALY averted) |
| 59 | 58 | 22 | 36 | 39772 | 2976 |
| 65 | 57 | 22 | 36 | 39793 | 3836 |
| 70 | 57 | 21 | 37 | 39809 | 4992 |
| 75 | 56 | 21 | 37 | 39825 | 7037 |
| 80 | 56 | 20 | 37 | 39840 | 11640 |
| 85 | 55 | 20 | 38 | 39855 | 31593 |
| 90 | 55 | 20 | 38 | 39869 | D |
| **Note:**  The comparison is between the five-yearly LBC combined with girl vaccination and five-yearly conventional cytology combined with girl vaccination.  D refers domination | | | | | |

**Figure 4: The incremental cost-effectiveness of liquid-based cytology compared to conventional cytology by different sensitivity of conventional cytology**

**Note:**

The screening is five-yearly

The strategies are combined with girl vaccination

The threshold of cost-effectiveness is 4822

**Figure 5: The probability of cost-effectiveness of five-yearly VIA combined with vaccination by cost of vaccine, screening and vaccination coverage.**

| **Note : By cost of vaccine and vaccination coverage, the combined strategy is compared to VIA alone. By screening coverage, the combined strategy is compared to vaccination alone.** |
| --- |

**Figure 6: The probability of cost-effectiveness of five-yearly conventional cytology combined with vaccination by cost of vaccine, screening and vaccination coverage.**

**Note : By cost of vaccine and vaccination coverage, the combined strategy is compared to conventional cytology alone. By screening coverage, the combined strategy is compared to vaccination alone.**

**Figure 7: The probability of cost-effectiveness of five-yearly combined VIA and cytology testing in addition to vaccination by cost of vaccine, screening and vaccination coverage.**

**Note : By cost of vaccine and vaccination coverage, the combined strategy is compared to combined VIA and cytology testing alone. By screening coverage, the combined strategy is compared to vaccination alone.**

**Figure 8: The probability of cost-effectiveness of five-yearly liquid-based cytology combined with vaccination by cost of vaccine, screening and vaccination coverage.**

**Note : By cost of vaccine and vaccination coverage, the combined strategy is compared to liquid-based cytology alone. By screening coverage, the combined strategy is compared to vaccination alone.**

**Figure 9: The probability of cost-effectiveness of five-yearly rapid HPV DAN testing combined with vaccination by cost of vaccine, screening and vaccination coverage.**

**Note : By cost of vaccine and vaccination coverage, the combined strategy is compared to rapid HPV DNA testing alone. By screening coverage, the combined strategy is compared to vaccination alone.**

**Reference**

1. Welcome to Lao Statistics Bureau. Vientiane Capital. Available: <http://www.nsc.gov.la/index.php?lang=en>. Accessed: 16 October 2014.

2. Bruni L, Barrionuevo-Rosas L, Serrano B, Brotons M, Cosano R, et al. Human Papillomavirus and Related Diseases in Laos. Summary Report 2014-03-17: ICO Information Centre on HPV and Cancer (HPV Information Centre). 2014. Available: http://www.hpvcentre.net/statistics/reports/LAO.pdf. Accessed: 09 February 2015.

3. Brennan A, Chick SE, Davies R. A taxonomy of model structures for economic evaluation of health technologies. Health Econ. 2006. 15: 1295-1310.

4. WHO/ICO Information Centre on HPV and Cervical Cancer (HPV Information Centre) (2012) Summary report on HPV and cervical cancer statistics in Laos 2010. Geneva: HPV Information Centre.

5. Canadian Agency for Drugs and Technologies in Health (cadth). Guidelines for the Economic Evaluation of Health Technologies: Canada. 3rd ed. Ottawa, ON: CADTH. 2006. 1-74 p.

6. Kim JJ, Andres-Beck B, Goldie SJ. The value of including boys in an HPV vaccination programme: a cost-effectiveness analysis in a low-resource setting. Br J Cancer. 2007. 97: 1322-1328.

7. Jit M, Choi YH, Edmunds WJ. Economic evaluation of human papillomavirus vaccination in the United Kingdom. BMJ. 2008. 337: a769.

8. Barnabas RV, Laukkanen P, Koskela P, Kontula O, Lehtinen M, et al. Epidemiology of HPV 16 and cervical cancer in Finland and the potential impact of vaccination: mathematical modelling analyses. PLoS Med. 2006. 3: e138.

9. Richart RM. A modified terminology for cervical intraepithelial neoplasia. Obstet Gynecol. 1990. 75: 131-133.

10. Gupta N, Srinivasan R, Rajwanshi A. Functional biomarkers in cervical precancer: an overview. Diagn Cytopathol. 2010. 38: 618-623.

11. Moscicki AB, Schiffman M, Kjaer S, Villa LL. Chapter 5: Updating the natural history of HPV and anogenital cancer. Vaccine. 2006. 24 Suppl 3: S3/42-51.

12. Borruto F, Comparetto C. Treatment, Follow-up, and Prevention of Papillomavirus Infection and Cervical Cancer. In: Borruto F, Ridder MD, editors. HPV and Cervical Cancer. New York: Springer Science+Business Media. 2012. pp. 273-318.

13. Holowaty P, Miller AB, Rohan T, To T. Natural history of dysplasia of the uterine cervix. J Natl Cancer Inst. 1999. 91: 252-258.

14. Benedet JL, Bender H, Jones H, 3rd, Ngan HY, Pecorelli S. FIGO staging classifications and clinical practice guidelines in the management of gynecologic cancers. FIGO Committee on Gynecologic Oncology. Int J Gynaecol Obstet. 2000. 70: 209-262.

15. Pecorelli S, Zigliani L, Odicino F. Revised FIGO staging for carcinoma of the cervix. Int J Gynaecol Obstet. 2009. 105: 107-108.

16. Lao statistics Bureau, Ministry of Health, UNICEF, UNFPA. Lao PDR: Lao Social Indicator Survey (LSIS). Vientiane, Lao PRD. 2012.

17. Sychareun V, Phengsavanh A, Hansana V, Chaleunvong K, Kounnavong S, et al. Predictors of premarital sexual activity among unmarried youth in Vientiane, Lao PDR: the role of parent-youth interactions and peer influence. Glob Public Health. 2013. 8: 958-975.

18. Bruni L, Barrionuevo-Rosas L, Serrano B, Brotons M, Cosano R, et al. Human Papillomavirus and Related Diseases in Thailand.: ICO Information Centre on HPV and Cancer (HPV Information Centre). 2014.

19. de Sanjose S, Quint WG, Alemany L, Geraets DT, Klaustermeier JE, et al. Human papillomavirus genotype attribution in invasive cervical cancer: a retrospective cross-sectional worldwide study. Lancet Oncol. 2010. 11: 1048-1056.

20. Kim JJ, Kuntz KM, Stout NK, Mahmud S, Villa LL, et al. Multiparameter calibration of a natural history model of cervical cancer. Am J Epidemiol. 2007. 166: 137-150.

21. Chen C, Yang Z, Li Z, Li L. Accuracy of several cervical screening strategies for early detection of cervical cancer: a meta-analysis. Int J Gynecol Cancer. 2012. 22: 908-921.

22. Suprasert P, Panyaroj W, Kietpeerakool C. Recurrent rates with cervical intraepithelial neoplasia having a negative surgical margin after the loop electrosurgical excision procedure in Thailand. Asian Pac J Cancer Prev. 2009. 10: 587-590.

23. WHO. Comprehensive cervical cancer control : a guide to essential practice. Switzerland: WHO. 2006. Available: <http://whqlibdoc.who.int/publications/2006/9241547006_eng.pdf>. Accessed 04 March 2013.

24. Arbyn M, Bergeron C, Klinkhamer P, Martin-Hirsch P, Siebers AG, et al. Liquid compared with conventional cervical cytology: a systematic review and meta-analysis. Obstet Gynecol. 2008. 111: 167-177.

25. Qiao L, Li B, Long M, Wang X, Wang A, et al. Accuracy of visual inspection with acetic acid and with Lugol's iodine for cervical cancer screening: Meta-analysis. J Obstet Gynaecol Res. 2015.

26. Chanthavilay P, Mayxay M, Phongsavan K, Marsden DE, White LJ, et al. Accuracy of Combined Visual Inspection with Acetic Acid and Cervical Cytology Testing as a Primary Screening Tool for Cervical Cancer: a Systematic Review and Meta-Analysis. Asian Pac J Cancer Prev. 2015. 16: 5889-5897.

27. Jeronimo J, Bansil P, Lim J, Peck R, Paul P, et al. A Multicountry Evaluation of careHPV Testing, Visual Inspection With Acetic Acid, and Papanicolaou Testing for the Detection of Cervical Cancer. Int J Gynecol Cancer. 2014. 24: 576-585.

28. Phongsavan K, Phengsavanh A, Wahlstrom R, Marions L. Safety, feasibility, and acceptability of visual inspection with acetic acid and immediate treatment with cryotherapy in rural Laos. Int J Gynaecol Obstet. 2011. 114: 268-272.

29. Macey R, George O, Zahnley T. Berkeley Madonna (Version 8.3.18). University of California. Available: http://www.berkeleymadonna.com/download.html. Accessed: 19 June 2013.

30. United Nations. Department of Economic and Social Affairs, Population Division (2013) World Population Prospects: The 2012 Revision, DVD Edition. Available: <http://esa.un.org/unpd/wpp/Excel-Data/population.htm>. Accessed: 12 July 2013.

31. PPP conversion factor, GDP (LCU per international $): World Development Indicators. World Bank, International Comparison Program database. Available: <http://data.worldbank.org/indicator/PA.NUS.PPP>. Accessed: 17 March 2015.

32. Shi JF, Chen JF, Canfell K, Feng XX, Ma JF, et al. Estimation of the costs of cervical cancer screening, diagnosis and treatment in rural Shanxi Province, China: a micro-costing study. BMC Health Serv Res. 2012. 12: 123.

33. Goldie SJ, O'Shea M, Campos NG, Diaz M, Sweet S, et al. Health and economic outcomes of HPV 16,18 vaccination in 72 GAVI-eligible countries. Vaccine. 2008. 26: 4080-4093.

34. Goldie SJ, Gaffikin L, Goldhaber-Fiebert JD, Gordillo-Tobar A, Levin C, et al. Cost-effectiveness of cervical-cancer screening in five developing countries. N Engl J Med. 2005. 353: 2158-2168.

35. WHO. 6th Global Meeting on Implementing New and Under-utilized Vaccines, 15-17 May 2012. New and Under-utilized Vaccines Implementation (NUVI): WHO. Available: <http://www.who.int/nuvi/2012_meeting_summary_introduction/en/index.html>. Accessed: 25 June 2014.

36. Johnson AM, Mercer CH, Erens B, Copas AJ, McManus S, et al. Sexual behaviour in Britain: partnerships, practices, and HIV risk behaviours. Lancet. 2001. 358: 1835-1842.

37. WHO. Life tables for WHO Member States. Geneva: World Health Organization. Available: <http://apps.who.int/gho/data/node.imr.LIFE_0000000029?lang=en>. Accessed: 06 March 2013.

38. Whitlock EP, Vesco KK, Eder M, Lin JS, Senger CA, et al. Liquid-based cytology and human papillomavirus testing to screen for cervical cancer: a systematic review for the U.S. Preventive Services Task Force. Ann Intern Med. 2011. 155: 687-697, W214-685.

39. Mitchell MF, Schottenfeld D, Tortolero-Luna G, Cantor SB, Richards-Kortum R. Colposcopy for the diagnosis of squamous intraepithelial lesions: a meta-analysis. Obstet Gynecol. 1998. 91: 626-631.

40. Sauvaget C, Muwonge R, Sankaranarayanan R. Meta-analysis of the effectiveness of cryotherapy in the treatment of cervical intraepithelial neoplasia. Int J Gynaecol Obstet 120: 218-223.

41. Jones HW, 3rd (1995) Cone biopsy and hysterectomy in the management of cervical intraepithelial neoplasia. Baillieres Clin Obstet Gynaecol. 2013. 9: 221-236.

42. Bosch FX. Human papillomavirus: science and technologies for the elimination of cervical cancer. Expert Opin Pharmacother. 2011. 12: 2189-2204.
